# Pleiotropic effects of PipX, PipY, or RelQ overexpression on growth, cell size, photosynthesis, and polyphosphate accumulation in the cyanobacterium *Synechococcus elongatus* PCC7942

**DOI:** 10.3389/fmicb.2023.1141775

**Published:** 2023-03-16

**Authors:** Antonio Llop, Jose I. Labella, Marina Borisova, Karl Forchhammer, Khaled A. Selim, Asunción Contreras

**Affiliations:** ^1^Departamento de Fisiología, Genética y Microbiología, Facultad de Ciencias, Universidad de Alicante, Alicante, Spain; ^2^Interfaculty Institute for Microbiology and Infection Medicine, Organismic Interactions Department, Cluster of Excellence 'Controlling Microbes to Fight Infections', University of Tübingen, Tübingen, Germany

**Keywords:** cyanobacteria, polyphosphate, stringent response, nitrogen regulation, PipX, PipY, (p)ppGpp

## Abstract

The cyanobacterial protein PipY belongs to the Pyridoxal-phosphate (PLP)-binding proteins (PLPBP/COG0325) family of pyridoxal-phosphate-binding proteins, which are represented in all three domains of life. These proteins share a high degree of sequence conservation, appear to have purely regulatory functions, and are involved in the homeostasis of vitamin B_6_ vitamers and amino/keto acids. Intriguingly, the genomic context of the *pipY* gene in cyanobacteria connects PipY with PipX, a protein involved in signaling the intracellular energy status and carbon-to-nitrogen balance. PipX regulates its cellular targets *via* protein–protein interactions. These targets include the PII signaling protein, the ribosome assembly GTPase EngA, and the transcriptional regulators NtcA and PlmA. PipX is thus involved in the transmission of multiple signals that are relevant for metabolic homeostasis and stress responses in cyanobacteria, but the exact function of PipY is still elusive. Preliminary data indicated that PipY might also be involved in signaling pathways related to the stringent stress response, a pathway that can be induced in the unicellular cyanobacterium *Synechococcus elongatus* PCC7942 by overexpression of the (p)ppGpp synthase, RelQ. To get insights into the cellular functions of PipY, we performed a comparative study of PipX, PipY, or RelQ overexpression in *S. elongatus* PCC7942. Overexpression of PipY or RelQ caused similar phenotypic responses, such as growth arrest, loss of photosynthetic activity and viability, increased cell size, and accumulation of large polyphosphate granules. In contrast, PipX overexpression decreased cell length, indicating that PipX and PipY play antagonistic roles on cell elongation or cell division. Since ppGpp levels were not induced by overexpression of PipY or PipX, it is apparent that the production of polyphosphate in cyanobacteria does not require induction of the stringent response.

## Introduction

Cyanobacteria, phototrophic prokaryotes that perform oxygenic photosynthesis, constitute a bacterial phylum with tremendous ecological impact on global carbon, nitrogen, and oxygen cycles, as they are the main contributors to marine primary production (Blank and Sánchez-Baracaldo, 2010). Their photosynthetic lifestyle and ease of cultivation prime them as ideal platforms for the sustainable production of high-value compounds, including biofuels (Khan and Fu, 2020). Cyanobacteria have evolved sophisticated systems to maintain the homeostasis of carbon/nitrogen assimilation (reviewed by Zhang et al., 2018; Forchhammer and Selim, 2020), the two most abundant elements in all living forms. Therefore, understanding the regulatory mechanisms controlling their metabolic balance is of paramount importance for environmental sustainability and biotechnological applications (Bougarde and Stensjö, 2022).

Cyanobacteria can use different nitrogen sources that are first converted into ammonium and then incorporated *via* the glutamine synthetase-glutamate synthase (GS-GOGAT) cycle into carbon skeleton 2-oxoglutarate (2-OG) for the biosynthesis of amino acids and other N-containing compounds (Muro-Pastor et al., 2005). The metabolite 2-OG, a universal indicator of the intracellular carbon-to-nitrogen balance (Senior, 1975; Huergo and Dixon, 2015), appears to be particularly suitable for this role in cyanobacteria (Forchhammer and Selim, 2020).

In bacteria and plants, 2-OG is sensed by the widely distributed and highly conserved homotrimeric signal transduction protein PII, encoded by *glnB*. PII regulates the activity of proteins involved in nitrogen and carbon metabolisms by direct protein–protein interactions (Selim et al., 2020; Forchhammer et al., 2022). The first PII receptors identified in cyanobacteria were detected in a search for proteins of the unicellular strain *Synechococcus elongatus* PCC7942 (hereafter *S. elongatus*) interacting with PII. One of them was PipX (PII interacting protein X), a small protein of 89 amino acids restricted to cyanobacteria (Burillo et al., 2004; Espinosa et al., 2006; Llácer et al., 2010; Labella et al., 2016, 2020a,b; Forcada-Nadal et al., 2018; Selim et al., 2019). The other one was *N*-Acetyl Glutamate Kinase (NAGK) (Llácer et al., 2007; Lapina et al., 2018).

PipX was also found as prey in yeast two-hybrid searches with the global transcriptional regulator NtcA, involved in nitrogen assimilation in cyanobacteria (Herrero et al., 2001; Esteves-Ferreira et al., 2018). PipX stabilizes the conformation of NtcA that is transcriptionally active and probably helps the local recruitment of RNA polymerase. Binding of PipX to PII or NtcA is antagonistically tuned by the 2-OG levels: Whereas high 2-OG levels favor the interaction of PipX with NtcA, they prevent the PipX-PII interaction. PII sequestration of PipX at low 2-OG renders PipX unavailable for NtcA binding and activation, reducing the expression of NtcA-dependent gene targets (Espinosa et al., 2007, 2009, 2010; Llácer et al., 2010; Zhao et al., 2010; Laichoubi et al., 2012). Moreover, the PipX-PII complex has an increased affinity for ADP, and therefore, the interaction between PII and PipX is highly sensitive to fluctuations in the ATP/ADP ratio, and thus to the energy state of the cells (Zeth et al., 2014; Espinosa et al., 2018; Selim et al., 2019).

Yeast three-hybrid searches with PipX–PII as bait resulted in the identification of the transcriptional regulator PlmA, a protein found exclusively in cyanobacteria (Labella et al., 2016). PlmA interacted with the PipX–PII complex but not with PII or PipX when provided independently and, thus emphasizing the regulatory potential of the PipX–PII complexes, the two hubs of the nitrogen interaction (Espinosa et al., 2018; Labella et al., 2020b).

Cyanobacterial genomes always contain at least as many copies of *glnB* as of *pipX* (Laichoubi et al., 2011), supporting the idea that a relatively high ratio of PII is needed to sequester free PipX and to counteract unwanted interactions with additional PipX partners. Indeed, excess PipX over PII prevents cell growth (Espinosa et al., 2007, 2009, 2010; Laichoubi et al., 2012; Chang et al., 2013; Jerez et al., 2021; Sakamoto et al., 2021), and so far the best candidate to mediate this response is the ribosome-assembly GTPase EngA, identified by synteny analysis (Labella et al., 2020a) and subsequently shown to interact with PipX (Jerez et al., 2021).

**Figure 1:**
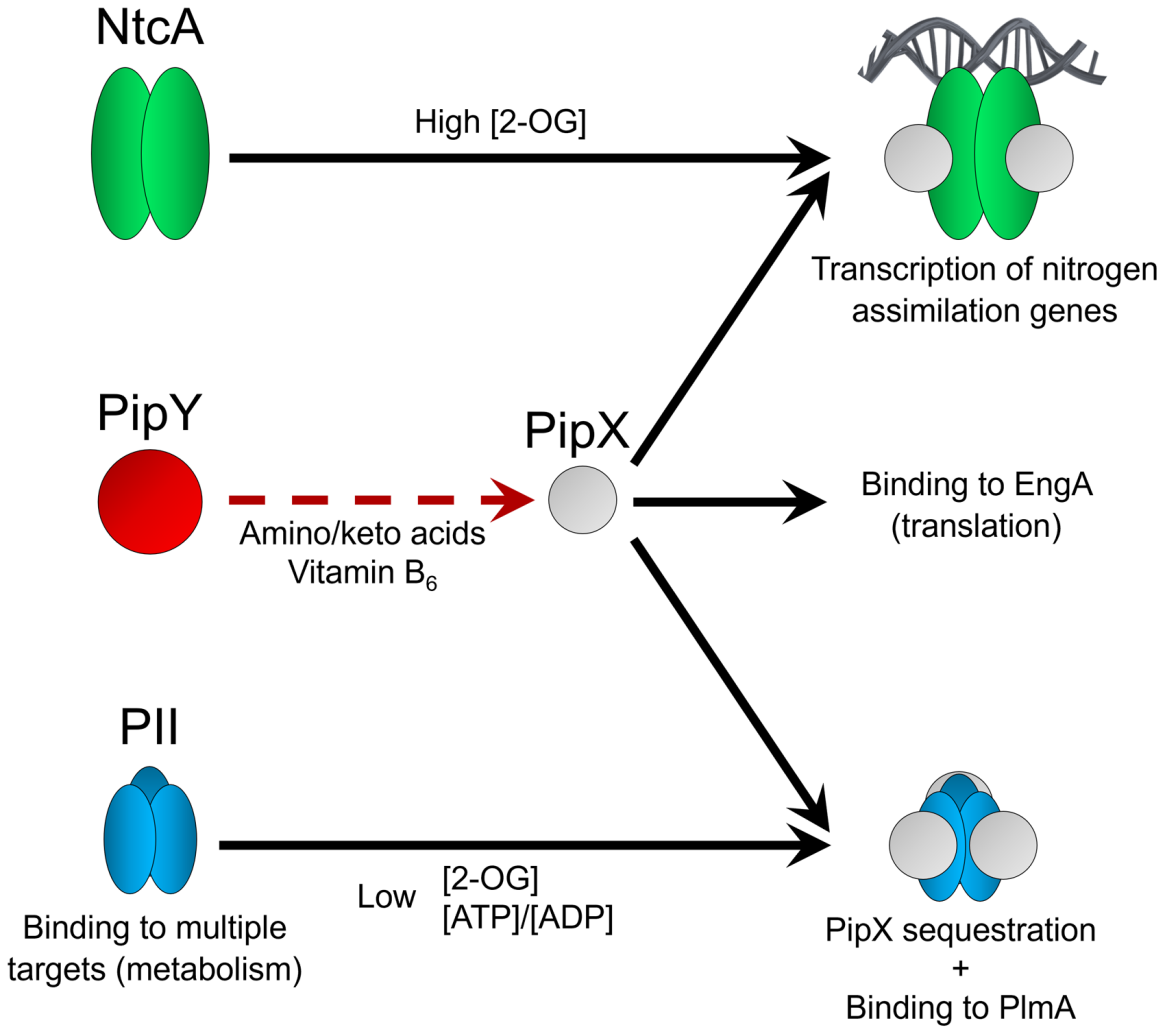
PipX swapping between PII and NtcA and putative role of PipY. The role of NtcA or PII effectors on interactions with PipX is illustrated with black solid arrows pointing toward the complexes. Functions of these and other PII or PipX complexes are mentioned below the corresponding complexes or proteins. Interactions between PipX and PipY, proposed from genetic evidence, is illustrated by the dotted red arrow. The area of the molecules is scaled according to the number of amino acids per protein.

Altogether, PipX swapping between PII and NtcA links PII signaling with NtcA-regulated gene expression, sequesters PipX to prevent unregulated interactions with additional partners and may also regulate PII availability for binding to target proteins involved in carbon and nitrogen metabolism (Figure 1).

In *S. elongatus pipX* forms a bicistronic operon with the downstream gene *pipY* (Labella et al., 2017), encoding a member of the widely distributed and highly conserved family of Pyridoxal-phosphate (PLP)-binding proteins (COG0325/PLPBP) that are involved in vitamin B_6_ and amino acid homeostasis (Ito, 2022; Tremiño et al., 2022). PLPBP deficiency altered the B_6_ pool in different organisms (Darin et al., 2016; Prunetti et al., 2016; Ito et al., 2019, 2020; Johnstone et al., 2019; Vu et al., 2020). Despite the obligate presence of the catalyst cofactor PLP in PipY and its orthologs, these proteins appear to have no enzymatic activity and are assumed to have purely regulatory functions (Tremiño et al., 2022). Since no direct protein targets or cellular components directly interacting with PLPBP family members have been reported to date, the operonic association of *pipY* with *pipX* provides a unique opportunity to investigate the roles of PLPBP members in the context of a signaling pathway highly conserved in cyanobacteria. In this context, it is remarkable that almost 80% of *pipX* genes are found adjacent to *pipY* genes, presumably as part of bicistronic *pipX*Y operons. Furthermore, the relatively short or non-existent intergenic distances found between contiguous *pipX* and *pipY* coding sequences are suggestive of tight co-regulation and translational coupling, thus supporting the idea of a functional interaction between PipX and PipY in cyanobacteria (Labella et al., 2017; Cantos et al., 2019), although the role played by PipY in the PipX interaction network and the molecular mechanisms involved remain to be established (Figure 1). PipY from *S. elongatus* is one of the best-characterized PLBPB members and can be regarded as a paradigm for these proteins (Labella et al., 2017; Tremiño et al., 2017, 2022; Cantos et al., 2019).

Inactivation of PLBPB members results in alterations of the relative levels of B_6_ vitamers and amino/keto acids in all organisms investigated (Ito, 2022; Tremiño et al., 2022). In *S. elongatus*, *pipY* null mutants display PLP-related phenotypes including increased sensitivity to pyridoxine and to the antibiotics D-cycloserine and β-chloro-D-alanine (Labella et al., 2017), in line with the importance of the PLP cofactor in PLPBP functions. Both PipX and PipY contribute to the expression levels of a common set of transcripts and the resistance to PLP-targeting antibiotics D-cycloserine and β-chloro-D-alanine, suggesting their involvement in the same, yet unknown, genetic pathway.

While null mutations at either *pipX* or *pipY* do not impair *S. elongatus* growth under standard laboratory conditions (Espinosa et al., 2009 and 2018; Labella et al., 2017), the so far obtained gain-of-function mutations at these genes are lethal and cannot be obtained in homozygosis (Laichoubi et al., 2012; data not shown).

Increasing the cellular abundance of PipY in *S. elongatus* augmented cell length (up to 28%), and we hypothesized that perturbations of the amino/keto acid pool by overexpression of PipY may result in the alteration of metabolic signals controlling cell size (Labella et al., 2017). Cellular metabolism is known to be a central regulator of the bacterial cell cycle (Sperber and Herman, 2017; Galinier et al., 2021) and, in line with this, a link between nutrient conditions and morphological flexibility has been established in *S. elongatus* (Goclaw-Binder et al., 2012). Interestingly, overexpressing the small (p)ppGpp (guanosine penta- and tetra-phosphate) synthetase from *Bacillus subtilis* YjbM/SAS1/RelQ (hereafter RelQ), also increased cell size in *S. elongatus* (Hood et al., 2016), raising the question of whether overexpression of PipY induce accumulation of the (p)ppGpp alarmone(s) and subsequently affect the cell size.

(p)ppGpp is the key signaling molecule of the stringent response, a global stress response program leading to the reorganization of gene expression that is broadly conserved in bacteria and plants. In most bacteria, nutrient limitation is the main stress inducing (p)ppGpp, whereas in cyanobacteria (p)ppGpp accumulates in response to the absence of photosynthetic activity such as during night periods (Hood et al., 2016; Puszynska and O’Shea, 2017). The enzyme mediating this response in cyanobacteria is the bifunctional (p)ppGpp synthetase/hydrolase Rel.

Non-diazotrophic cyanobacteria have a specific stress program induced by nutrient starvation termed chlorosis or bleaching, a complex adaptive response by which they degrade their light-harvesting antenna, the phycobilisome, and reduce their chlorophyll content (Schwarz and Grossman, 1998; Forchhammer and Schwarz, 2019). Nitrogen deprivation is the most dramatic trigger of chlorosis, but chlorosis can also be induced in *S. elongatus* by overexpression of PipX (Jerez et al., 2021), RelQ (Hood et al., 2016) or PipY (this work).

To gain insights into the functions of *S. elongatus* PipY and its regulatory connections with PipX, we now analyze in parallel the phenotypic effects of overexpressing PipX, PipY, and RelQ. We have found that very high levels of PipY severely impair cell viability and photosynthesis and produce a dramatic and unprecedented accumulation of giant polyphosphate (hereafter polyP) granules without increasing the levels of ppGpp. We also found that the intracellular accumulation of PipX decreases cell length, a result implying that at high intracellular levels, PipX and PipY have opposite effects on the same trait.

## Materials and methods

### Cyanobacterial strains and growth conditions

*Synechococcus elongatus* strains and plasmids used in this work are described in Table 1. Strains were grown in BG11 media [BG11_0_ plus 17.5 mM sodium nitrate (NaNO_3_) and 10 mM HEPES/NaOH, pH 7.8; Rippka et al., 1979], except for nitrogen deprivation (BG11_0_) or varying dipotassium phosphate levels [K_2_HPO_4_ 0.11 mM (0.5×) or 0.44 mM (2×)]. Cultures were routinely grown photo-autotrophically under constant illumination provided by cool white fluorescent lights at 30°C in baffled flasks (shaking: 150 rpm, 70 μmol photons m^−2^ s^−1^) or on plates (30 μmol photons m^−2^ s^−1^). For growth on plates, media was solidified with 1.5% (w/v) agar, with the addition of 0.5 mM sodium thiosulfate (Na_2_S_2_O_3_) after autoclaving. To overexpress proteins 50 μM of isopropyl β-D-1-thiogalactopyranoside (IPTG) was added to media. To select genetically modified strains, the antibiotics chloramphenicol (chl; 3.5 μg mL^−1^), streptomycin (str; 6 μg mL^−1^), or kanamycin (kan; 12 μg mL^−1^) were added.

**Table 1:** Plasmids and strains used in this work.

Plasmid or strain	Genotype, relevant characteristic	Source or references
Plasmid		
pUAGC126	*pipX* replaced with *cat*, amp^R^, chl^R^	Labella et al. (2017)
pUAGC127	*pipY* replaced with *cat,* amp^R^, chl^R^	Labella et al. (2017)
pUAGC280	NSI, *lacI* Ptrc, amp^R^, str^R^	Moronta-Barrios et al. (2013)
pUAGC294	NSI, *lacI* Ptrc::*pipY,* amp^R^, str^R^	Labella et al. (2017)
pUAGC873	NSI, *lacI* Ptrc::*pipX,* amp^R^, str^R^	Labella et al. (2017)
pSAV061	NSII, *lacI* Ptrc::*relQ,* amp^R^, kan^R^	Hood et al. (2016)
Strain		
WT	*Synechococcus elongatus* PCC7942	Pasteur culture collection
*pipX*	PipX^−^, Δ*pipX::cat*, chl^R^	Labella et al. (2017)
*pipY*	PipY^−^, Δ*pipY::cat*, chl^R^	Labella et al. (2017)
1^S^Ptrc	ɸ(NSI-P_trc_), str^R^	Moronta-Barrios et al. (2013)
1^S^Ptrc-PipX	PipX^ox^, ɸ(NSI-P_trc_::*pipX*), str^R^	Labella et al. (2017)
1^S^Ptrc-PipY	PipY^ox^, ɸ(NSI-P_trc_::*pipY*), str^R^	Labella et al. (2017)
2^K^Ptrc-RelQ	RelQ^ox^, ɸ(NSII-P_trc_::*relQ*), kan^R^	Hood et al. (2016)

Amp, ampicillin; Cat, chloramphenicol acetyltransferase.

To obtain strain derivatives, *S. elongatus* was transformed with the corresponding plasmids essentially as described by Golden and Sherman (1984) and complete segregation was analyzed by PCR. To confirm allele replacement at *pipX* or *pipY* loci, the following pairs of primers were used: PipX-126-F (5′-TAAAAACTAGCCGCCCTTGC-3′) and PipX-5R-X (5′-CAGCCCGCAAATCAGCAG-3′), or inter2060-1F (5′-GATCGGAATTCCCAGCTAGGGAGTCAGAGG-3′) and PipX-5R-X (5′-CAGCCCGCAAATCAGCAG-3′), respectively. To verify sequences within NSI or NSII, the following pairs of primers were used: NSI-1F (5′-CGACATCTTCCTGCTCCAG-3′) and NSI-1R (5′-TGCCTGAAAGCGTGACGAGC-3′) for 1^S^Ptrc, 1^S^Ptrc-PipX, and 1^S^Ptrc-PipY strains, or NSII-1F (5′-AGGTTGTCCTTGGCGCAGCG-3′) and NSII-1R (5′-AGCGGATTTTGCATCACGAAGC-3′) for strain 2^K^Ptrc-RelQ.

For growth measurements in flasks experiments, 30 mL of cultures was adjusted to an initial optical density at 750 nm (OD_750_) of 0.1 using a Ultrospec 2,100 pro UV–Vis Spectrophotometer (Amersham) and then recorded at times 0, 24, and 48 h after the addition of IPTG. For constant measurements, 70 mL of cultures was grown in a Multi-cultivator OD-1000 with a Gas Mixing System GMS 150 (Photosystems Instruments, Drasov, Czech Republic), supplied with air under constant illumination at 50 μmol m^−2^ s^−1^. Optical density was determined at 720 nm (OD720) with an interval of 1 h until 142 h. Doubling time was calculated using the data from the control strain (1^S^Ptrc) for the time range 0–48 h with a free online doubling time calculating software.^1^

For drop-plate assays, 5 μL of culture samples adjusted to an OD_750_ of 0.1 was spotted on BG11 plates. To test the loss of sensitivity to IPTG of previously induced cultures, streptomycin- (str^R^; PipX^ox^ or PipY^ox^) or kanamycin-resistant (kan^R^, RelQ^ox^) colonies were selected and subsequently replicated to plates with or without IPTG.

To follow the dynamics of polyP granules formation during diurnal cycles, cultures were grown for 3 days in 12 h light/12 h darkness cycles until they reach an OD_750_ of 0.5–0.6.

### Determination of pigment content, apparent photosystem II (PSII) quantum yield and oxygen evolution

Whole-cell absorbance spectra (350–750 nm) were determined on a UV/Visible Ultrospec 3,100 pro (Amersham) in 1 mL of cultures previously adjusted to an OD_750_ of 0.4–0.5. Pigment content absorbance maxima at 635 and 685 nm corresponded to phycocyanin and chlorophyll *a*, respectively (Poniedziałek et al., 2017). Data were normalized by the OD_750_ of each strain at the different timepoints. To quantify the *in vivo* apparent PSII quantum yield, 100 μL of the previously adjusted samples (light adapted) was diluted 1:20 with 1.9 mL of H_2_O and measurements were carried out in a WATER-PAM chlorophyll fluorometer (Walz GmbH) as described previously (Selim et al., 2018, 2021). The maximal PSII quantum yield (F_v_/F_m_) was determined with the saturation pulse method. ΔF yield indicates the maximal PSII quantum yield (F_v_/F_m_) of three measurements of each sample.

To evaluate the oxygen evolution, 1 ml of cultures was adjusted to an OD_750_ of 0.5. Measurements were taken at room temperature using a Clark-type oxygen electrode DW1 during a 300/300 s light/dark to follow the release and consumption of oxygen, respectively. The light (50 μmol photons m^−2^ s^−1^) was provided from a high-intensity white light source LS2 (Hansatech). Data were normalized by the initial measure (in light) for each strain.

### Cell staining, confocal microscopy, determination of cell lengths, and polyphosphate measurements

Detection of polyP granules with 4′,6-diamidino-2-phenylindole (DAPI) in *S. elongatus* was based on a previously published protocol (Hood et al., 2016). 1 mL of samples was fixed with 1% formaldehyde for 20 min at room temperature (RT), washed once with PBS 0.25× and frozen at −20°C for 24 h. Staining was carried out with 0.5 mg mL^−1^ of DAPI, 15 min in the dark at room temperature, followed by three washing steps with PBS 0.25×.

Bis-(1,3-Dibutylbarbituric Acid)-trimethine oxonol (DiBAC4(3)) was used as described in (Doello et al., 2021). 190 μL of cultures at timepoints 24 and 48 h after IPTG induction was stained for 30 min in the dark at RT with 10 μM DiBAC4(3) dissolved in dimethyl sulfoxide (DMSO). Dead cells were obtained by boiling the sample at 99°C for 10 min.

Black-white micrographs were taken on a confocal laser scanning microscope Zeiss model LSM800 by dropping 5 μL of cultures on 2% low-melting-point agarose pads. Microscopic images were colored as red (auto-fluorescence), light-blue (polyP), and yellow (DiBAC4(3)) to improve visualization and contrast. The filter parameters were as follows: bright-field with transmitted light for cell visualization; Ex 640 nm/Em 650+ nm for cyanobacterial auto-fluorescence analysis; and Ex 405 nm/Em 470–617 nm for polyP; Ex 488 nm/Em 520–550 nm for DiBAC4(3).

The ImageJ software was used to determine cell length of the confocal microscopy auto-fluorescence images. Measurements were performed manually due to the significant differences in cell auto-fluorescence and morphology among the compared samples. The length was determined with the “Straight” tool of the program and the “Measure” button. Two hundred cells from two biological replicates were measured for each strain in each condition. The boxplot representation and Wilcoxon rank sum test analysis of cell length and PAM data were performed with the RStudio program (RStudio Team, 2020).

Automatic detection and measurement of polyP granules was carried out using a custom python script relying on the open-cv2 library. First, cells were detected in the blurred (Gaussian blur with a (5, 5) kernel), thresholded (using the thresh binary option with a minimum value set by hand for each micrograph), and eroded [(5,5) kernel, 2 iterations] autofluorescent signal micrographs using the findContours function. For each detected contour, cell area (obtained with the contourArea function applied to the contour) was compared to the hull area (obtained with the contourArea function applied to the result of the convexHull function applied to the contour). Contours with the hull area equal or less than 105% of the contour area were labeled as single cell contours. PolyP frequency per cell was only calculated in these single-cell contours. For polyP detection, each detected cell contour was used as a mask to obtain the region of interest out of the DAPI signal micrograph where polyPs can be observed. The generated image was blurred [Gaussian blur (5,5) kernel] and the median pixel value was obtained. The image was thresholded using twice the median value as a threshold, and polyP was detected using the findContour function. For each polyP contour, its area was calculated with the contourArea function. The number of polyP contours inside cells was recorded only at single-cell contours.

### Transmission electron microscopy

1.8 mL of culture samples was adjusted to 0.5 OD_750_ and fixed for 10 min at room temperature with 0.2 mL of 25% glutaraldehyde (4°C overnight incubation). Then, precipitated cells were carefully washed 3 times with 5 mM HEPES buffer pH 8, and resuspended in 500 μL of 2% potassium permanganate (KMnO_4_), incubated overnight at 4°C. After washing 5 to 8 times with H_2_O, samples were dehydrated with crescent ethanol (C_2_H_6_O) concentrations (from 70 to 100%) and embedded in EMbed-812 (Epon-812 substitute from Electronic Microscopic Science). Ultrathin sections were stained with uranyl acetate and lead citrate and cells visualized with a Philips Tecnai electron microscope at 80 kV.

### *In vivo* nucleotide extraction and mass spectrometry analysis

Nucleotide isolation was based on a previously published protocol by Juengert et al. (2017). IPTG was added to 400 ml of cultures at an initial OD_750_ of 0.4. At timepoints 0, 12, and 24 h, the same amount of cells (≤50 mL of cultures, a total of 20 OD_750_) were collected on glass microfiber filters (Whatman^®^, cat. Number 0.45 μm pore size, *d* = 47 mm). Filters were immediately transferred into 50 mL falcon tubes, flash-frozen in liquid nitrogen and stored at −80°C. For the extraction of nucleotides and ppGpp, the falcon tubes with the frozen cells were placed on ice and the cells were immediately resuspended in 3 mL of 2 M formic acid. Samples were then incubated on ice for 30 min and the cells were further disrupted using 0.1 mm zirconia/silica beads with 6 cycles of 30/30 s, a speed of 5 m/s and at 4°C in a high-speed homogenizer FastPrep^®^-24 Ribolyser (MP Biomedicals). The cells were pelleted by centrifugation (3 min, 16,000 × *g*, 4°C) and the aqueous phase was transferred to 3 mL of 50 mM ammonium acetate (NH_4_OAc) pH 4.5. Oasis WAX 1 cc Vac cartridges (60 mg sorbent per cartridge; particle size, 30 μm) were equilibrated with 3 mL CH_3_OH (analytical grade) and then with 3 mL of 50 mM ammonium acetate using centrifugation (5 min, 5,000 rpm, 4°C) for the elution. The samples were loaded into the equilibrated cartridges and afterward, the cartridges were washed with 3 mL 50 mM ammonium acetate pH 4.5 and then with 3 mL methanol (CH_3_OH). Each sample was eluted by centrifugation with 1 mL of CH_3_OH:ddH_2_O:NH_4_OH = 20:70:10 and the solutions were lyophilized overnight at RT. Nucleotide-enriched lyophilized samples were resuspended in 100 μl ddH_2_O prior to the HPLC-MS analysis.

Nucleotide analysis by HPLC-MS was performed as previously described with some modifications (Kästle et al., 2015) using an ESI-TOF mass spectrometer (micrO-TOF II, Bruker) operated in negative-ion mode and connected to an UltiMate 3,000 high-performance liquid chromatography (HPLC) system (Dionex). 5-μL of each sample (stored at 10°C in the autosampler) was injected onto the SeQuant ZIC-pHILIC column (Merck, PEEK 150 × 2.1 mm, 5 μm), and the sample separation was performed at 30°C as using a 35-min gradient program and a flow rate of 0.2 mL/min: 5 min of 82% buffer A [Acetonitrile (CH_3_CN)] and 18% buffer B [100 mM ammonium carbonate ((NH_4_)_2_CO_3_) pH 9.2]; 20 min of a linear gradient to 58% buffer B; and finally, 10 min of 82% buffer A. The exact masses in the negative ion mode for ppGpp of (M-H)^−^ = 601.9497 m/z and for phosphoric acid (H_3_PO_4_) of (M-H)^−^ = 96.9696 m/z were calculated in the program Mass Calculator (GitHub—axelwalter/MassCalculator). MS data obtained from micrO-TOF II were converted to mzML format and analyzed in Easy-MS program (GitHub— axelwalter/easy-MS) by calculating the area under the curve (AUC) of the extracted ion chromatograms for ppGpp and for phosphoric acid, using an AUC baseline, respectively, of 100 and 200. Data were presented as AUC of the extracted ion chromatograms for ppGpp normalization to the AUC of the extracted ion chromatograms for phosphoric acid.

## Results and discussion

### Distinctive features of growth arrest induced by overexpression of PipX, PipY or RelQ

*Synechococcus elongatus* strains 1^S^Ptrc-PipX, 1^S^Ptrc-PipY, 2^K^Ptrc-RelQ, or 1^S^Ptrc (Table 1) designed for inducible overexpression of PipX (synpcc7942_2061), PipY (synpcc7942_2060), RelQ (BSU11600), or no protein (control) were obtained by allelic replacement after transformation with plasmids pUAGC873, pUAGC294, pSAV061 or pUAGC280 (Table 1). In these strains, transcription of the corresponding genes takes place from the same IPTG-inducible promoter (P*trc*), within cassettes flanked by neutral sites NSI or NSII to provide recombination sites at the *S. elongatus* chromosome. The cassettes also encode a selection marker [streptomycin (str) or kanamycin (kan)] and the LacI repressor for control of gene expression (see details in Supplementary Figure S1). For simplicity, we will hereafter refer to the IPTG-induced cultures, cells, strains, or extracts of 1^S^Ptrc-PipX, 1^S^Ptrc-PipY, 2^K^Ptrc-RelQ, and 1^S^Ptrc as PipX^ox^, PipY^ox^, RelQ^ox^, and Ptrc, respectively. Overexpression experiments described hereafter were always carried out in IPTG-containing, antibiotic-free media.

**Figure 2:**
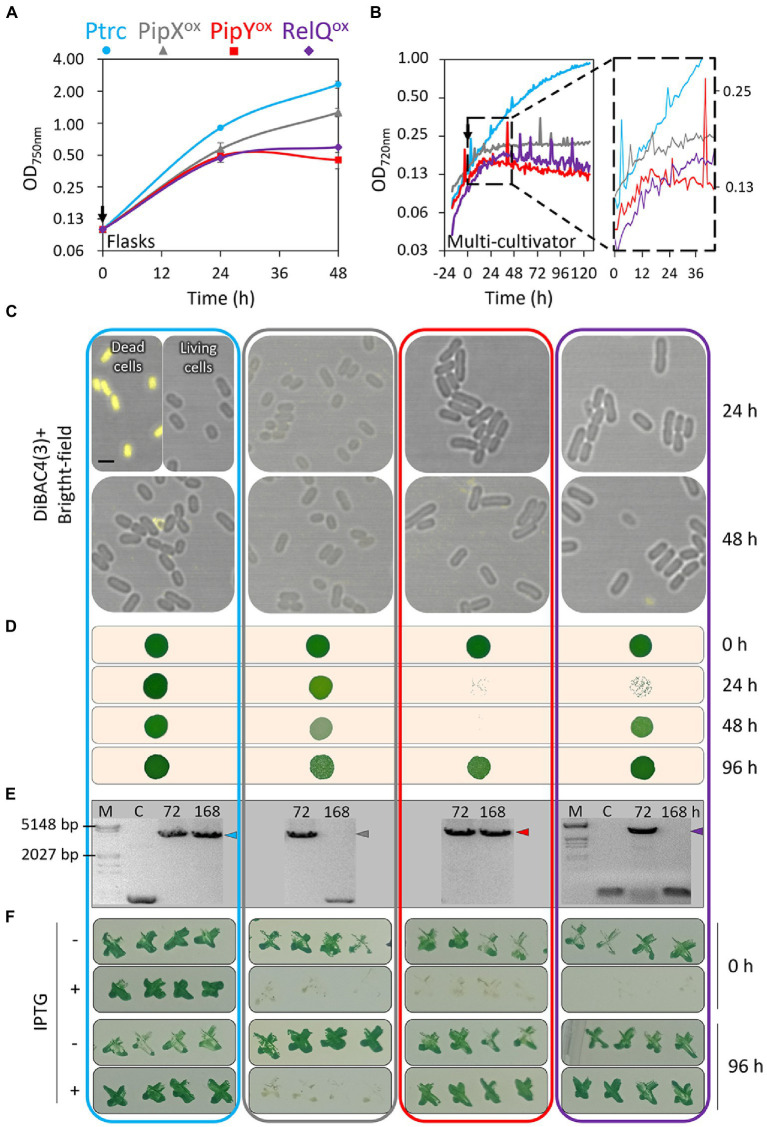
Growth-related phenotypes in cultures overexpressing PipX, PipY, or RelQ. Growth curves of the indicated strains in standard culture conditions (BG11 media) with IPTG, measured **(A)** at OD 750 nm from flasks or **(B)** at OD 720 nm from a multi-cultivator. The zoom at the inset covers corresponds to the 30 h following IPTG addition, indicated by a black arrow. Data are presented as mean and error bars (standard deviation) of two independent experiments. **(C)** Representative confocal micrographs showing the overlay of DiBAC4(3) staining (Ex 490–510 nm) and bright-field of cells after 24 or 48 h of IPTG induction. “Dead cells” (only for Ptrc) had been heated for 10 min at 99°C before staining. Scale bar, 2 μm. **(D)** Drop-plate assay (5 μL; 0.1 OD_750_) on IPTG-free medium from IPTG-containing cultures from the indicated timepoints. **(E)** PCR analyses to verify the stability of overexpression constructs from IPTG-containing cultures from the indicated timepoints. In each gel the colored arrow indicates the band size characteristic of each strain. Reference size bands (numbers) are indicated to the left. M, marker; C, WT control strain. PCR and running conditions were different only for the RelQ^ox^ panel. See Supplementary Figure S1 for additional details. **(F)** Growth with or without IPTG of crosses from single antibiotic-resistant colonies isolated from cultures at the indicated timepoints of the IPTG treatment. IPTG was always used at 50 μM. Plate photographs were taken after 5 days. Labels, data points, and panel outlines are colored to indicate the strains as follows: Ptrc-light blue, PipX^ox^-gray, PipY^ox^-red, RelQ^ox^-purple.

The impact of IPTG addition on the growth of all four strains was first followed using standard flasks with shaking. As shown in Figure 2A, a greater impact on culture growth was observed for PipY^ox^ and RelQ^ox^ than for PipX^ox^. In comparison, PipY^ox^ appears to be slightly more affected than RelQ^ox^ at the end of the experiment, a tendency observed in independent experiments (Supplementary Figure S2). It is worth noting that after 4 days of IPTG treatment, all three cultures start to attenuate their chlorotic appearance and after 5–6 days they had increased density and recovered their characteristic green color (Supplementary Figure S3), indicating that, in these antibiotic-free cultures, phenotypically different cells proliferate under prolonged treatment with IPTG (see below).

To perform more detailed growth analysis, particularly at times closer to the addition of IPTG, we repeated the experiment using a multi-cultivator where cells grow more slowly (doubling time 24.75 h versus just 7.56 h during the exponential phase in flasks) and importantly, continuous measurement of the optical density is obtained. As shown in Figure 2B, the previously observed effects of IPTG on the growth of the different strains were all confirmed. Importantly, continuous measurement of the optical density revealed that growth retardation of PipY^ox^ and PipX^ox^ strains started earlier (at about 10–12 h) than in RelQ^ox^ cultures (stationary after 24 h). Thus, the relatively early growth-arrest responses of PipY^ox^ and PipX^ox^ cultures argue in favor of the overexpressed proteins having direct inhibitory effects on *S. elongatus* growth.

### Overexpression of PipY or RelQ, but not of PipX, impairs recovery and favors IPTG resistant mutants

To investigate whether the observed growth arrest involves increased lethality, we stained PipX^ox^, PipY^ox^ and RelQ^ox^ cells with the fluorescent membrane potential reporter bis-(1,3-dibutylbarbituric acid)-trimethine oxonol [DiBAC4(3)], using heat-treated Ptrc cells as control for depolarized dead cells. As shown in Figure 2C, no apparent differences were found between the Ptrc control and PipX^ox^, PipY^ox^, or RelQ^ox^ cells, indicating that they all maintained their membrane potential after 24–48 h of IPTG induction. However, the DiBAC4(3) assay does not detect possible differences in cell viability and we wondered about the ability of the IPTG-treated cultures to regain growth on permissive conditions, that is, after transfer to fresh media (without added IPTG). With this in mind, we performed drop-plate assays to compare the ability of the different IPTG-treated cultures to recover growth in the absence of IPTG (Figure 2D).

After 24, 48, or 96 h of induction, drops from PipX^ox^ cultures produced spots that were just slightly less dense than those of the Ptrc control, in agreement with our previous data (Jerez et al., 2021). The fact that the toxic effect of PipX overexpression is reversed by removal of the IPTG inducer indicates a rapid turn-over of the PipX protein: As soon as overexpression of PipX is stopped, PipX homeostasis is re-established. The inhibitory role of excess levels of PipX on cell growth can be easily reconciled with the known roles of PipX at increasing NtcA transcriptional activity (Espinosa et al., 2007, 2014) and/or at interfering with the function of the ribosome-assembly GTPase EngA (Jerez et al., 2021).

Interestingly, only a few individual colonies were obtained on the spots from PipY^ox^ and RelQ^ox^ cultures at the 24 h timepoint, indicating that most of the cells from these cultures had already lost the ability to grow under permissive conditions. However, very different results were observed for PipY^ox^ and RelQ^ox^ after 48 h of induction, with almost undetectable colonies in the case of PipY^ox^ and a continuous lawn in the case of RelQ^ox^. Since later on both cultures produced continuous lawns (96 h timepoint), it appeared that subpopulations of actively growing cells took over the cultures and that the fast-growing cells appeared earlier in RelQ^ox^ than in PipY^ox^ cultures, probably reflecting different frequencies for suppressor mutations.

To explore the stability of the overexpression constructs, PCR amplification of the NSI or NSII sites from Ptrc, PipX^ox^, PipY^ox^, or RelQ^ox^ cultures under prolonged IPTG exposure (in the absence of antibiotics) was carried out. The presence of small bands matching the sizes of the corresponding control alleles NSI or NSII instead of the longer bands from the overexpression constructs in PipX^ox^ or RelQ^ox^ (Figure 2E) suggested the instability of the corresponding alleles under prolonged IPTG pressure.

To independently validate the results obtained here and to gain further insights into the overexpressing strains, we next reobtained 1^S^Ptrc, 1^S^Ptrc-PipX, 1^S^Ptrc-PipY, and 2^K^Ptrc-RelQ strains and treated them with IPTG for up to 96 h. Individual colonies retaining the cassette resistance-marker were obtained from all four cultures at the start and at the end of the experiment and in each case, these str^R^ or kan^R^ colonies were tested for IPTG sensitivity on plates. Note that by focusing on the colonies retaining the selection marker we were ignoring the potentially abundant but less informative mutants that eliminate (at least partially) or significantly re-arrange the overexpression cassettes. Representative results for each of the strains are shown in Figure 2F.

The finding that, up to 96 h under IPTG treatment, all (str^R^) PipX^ox^ clones tested remained IPTG sensitive suggest that most, if not all, of the fast growing cells in those cultures were str^S^. In sharp contrast, all (str^R^) PipY^ox^ or (kan^R^) RelQ^ox^ were as resistant to IPTG after prolonged culture with IPTG as the Ptrc control, suggesting that suppression of growth arrest may be caused by mutations outside the overexpression cassettes. Therefore, it appears that it is easier to suppress PipY^ox^ or RelQ^ox^ growth defects than PipX^ox^. Our interpretation of these results is that mutations suppressing PipY^ox^ or RelQ^ox^ (but not PipX^ox^) growth defects may be loss-of-function mutations at *S. elongatus* chromosomal genes while reverting PipX^ox^ cells to fast growth would not involve loss-of-function mutations at *S. elongatus* genes but rather specific mutations, most likely at an essential gene.

Suppressors of the growth phenotypes caused by overexpression of PipX, PipY, or even RelQ, a heterologous gene from *B. subtilis*, are of great interest as they can be used to identify cell components functionally interacting with PipX or PipY in *S. elongatus*, or to reveal targets and effectors participating in the stringent response. Since the mechanisms of transcriptional control exerted by (p)ppGpp are fundamentally different in the two well characterized bacterial systems *B. subtilis* and *E. coli* and none of the known strategies appear to be used by *S. elongatus* (Nomura et al., 2014; Hauryliuk et al., 2015), the molecular targets of t(p)ppGpp in cyanobacteria remain so far enigmatic.

### Distinctive features of chlorosis induced by overexpression of PipX, PipY, or RelQ

**Figure 3:**
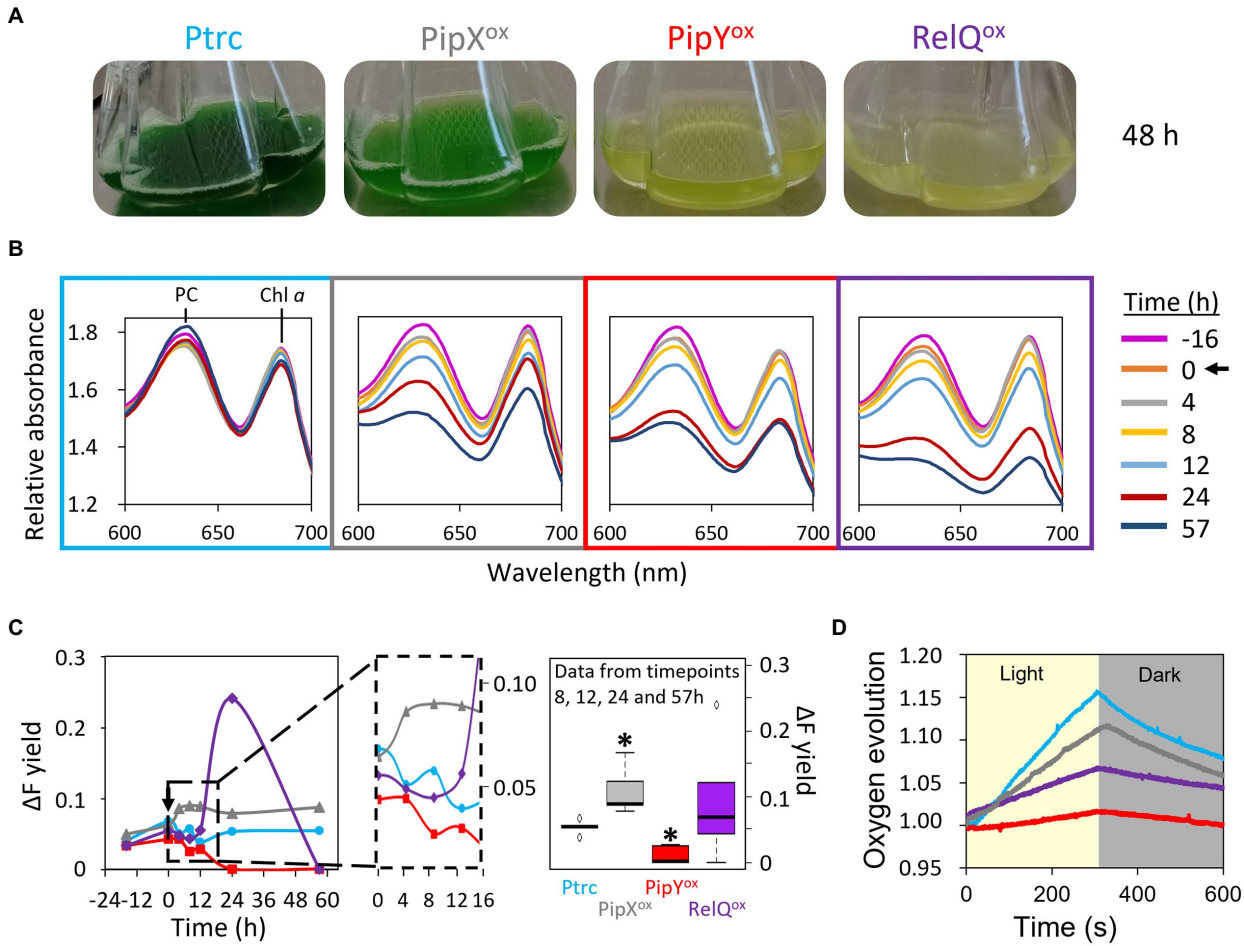
Distinctive features of chlorosis induced by PipX^ox^, PipY^ox^, or RelQ^ox^. **(A)** Visual appearance of cultures 48 h after IPTG addition. **(B)** Whole absorbance spectra of cultures treated with IPTG for the indicated times. Phycocyanin (PC) and chlorophyll *a* (Chl *a*) peaks are indicated. Data representative from one out of three independent experiments. **(C)** (left) Apparent PSII quantum yield (ΔF yield) measured by PAM fluorometry (one out of two representative experiments) and (right) boxplots of data from the timepoints 8, 12, 24, and 57 h. The box of two independent experiments contains 50% of the values, with median indicated as a horizontal line and dots representing the outliers. Wilcoxon rank sum test analysis of data between Ptrc and overexpression strains produced *p*-values <0.01 (*). **(D)** Oxygen evolution during a 300/300 s light/dark exposure of strains treated with IPTG for 24 h. In **(B,C)** black arrows indicate the exact timepoint of IPTG addition, and the inset corresponds to zoom of the indicated region. Labels, data points, and panel outlines are colored to indicate the strains as follows: Ptrc-light blue, PipX^ox^-gray, PipY^ox^-red, RelQ^ox^-purple. Other details as in Figure 2.

To get further insights into the physiological effects of PipX or PipY overexpression, we next analyzed in more detail the loss of pigmentation of PipX^ox^, PipY^ox^, and RelQ^ox^ cultures upon IPTG induction. Loss of pigmentation, degradation of light-harvesting phycobilisome, and substantial loss of thylakoid membranes are a hallmark of chlorosis, a process that naturally occurs in wild-type cells in response to nutrient starvation (Forchhammer and Schwarz, 2019 and Supplementary Figure S4). The results of the analyses concerning photosynthesis parameters are summarized in Figure 3.

**Figure 4:**
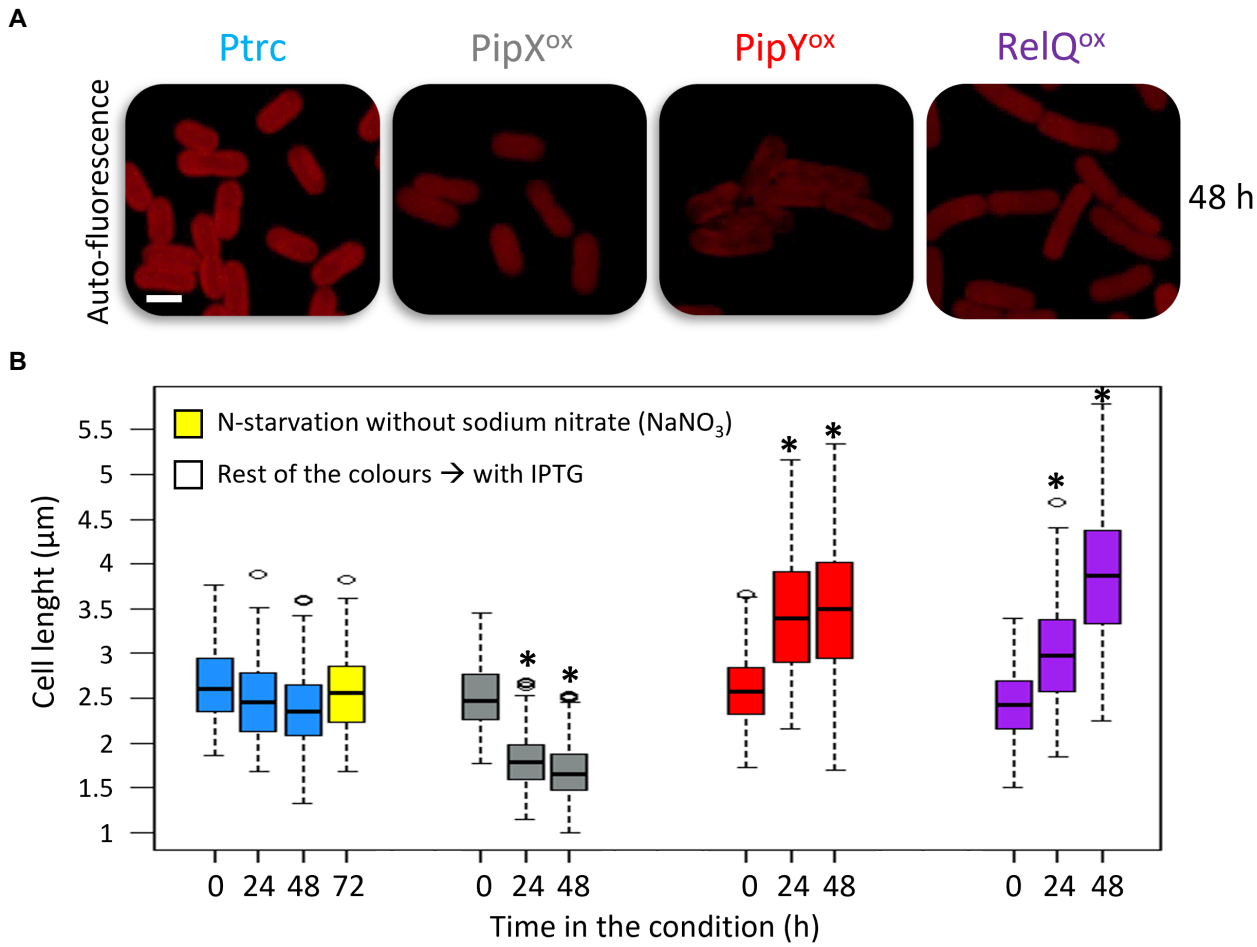
PipX^ox^ has opposite effects on cell length than PipY or RelQ. **(A)** Confocal micrographs showing the auto-fluorescence signal (Ex 633 nm) after 48 h and **(B)** cell-length boxplots of cells grown in BG11 with IPTG for the indicated number of hours. Scale bar, 2 μm. The median, the box from the first to the third quartile, and the outliers (dots) are represented. The number of cells measured from two biological replicates was *n* = 200. Wilcoxon rank sum test analysis of cell length data between Ptrc and overexpression strains in each timepoint produced *p*-values <2.2e-16 (*). Labels and boxplots are colored to indicate the strains as follows: Ptrc-light blue, PipX^ox^-gray, PipY^ox^-red, RelQ^ox^-purple, Ptrc without nitrogen-yellow. Other details as in Figure 2.

In all three cases, pigment loss could be detected after 8–12 h of the IPTG induction, although PipX^ox^ bleached significantly more slowly (Figures 3A,B). RelQ^ox^ reached the lowest levels of Chl *a* and, even more obvious, of phycocyanin at the end of the 57 h experiment. In close agreement with the chlorotic appearance of cultures, confocal microscopy after 48 h of IPTG induction showed a clear reduction in the autofluorescence of PipY^ox^ and RelQ^ox^, and to a lesser extent of PipX^ox^ (Figures 3A, 4A). Therefore, for all three overexpressing cultures analyzed here we observed a good correlation between the severity of growth phenotypes and bleaching. In this context, it is worth noting that the bleaching of cultures is not necessarily associated with growth arrest. For instance, *S. elongatus* cultures subjected to relatively high intensities of light become yellowish while growing at least at fast as the control under standard light conditions (Supplementary Figure S5).

Next, we analyzed the dynamics of photosynthesis parameters. When the apparent photosystem II (PSII) quantum yield was characterized using pulse amplitude-modulated (PAM) fluorometry, all three overexpressing strains behaved differently to Ptrc as well as to each other (Figure 3C). PipY^ox^ and RelQ^ox^ showed opposite responses soon after the IPTG induction (4–8 h), reaching maximal differences after 24 h, when PSII quantum yield became undetectable for PipY^ox^. The pronounced increase in the PSII quantum yield of RelQ^ox^ upon induction was followed by an also fast decrease, decaying to undetectable levels after 2 days of the IPTG addition (57 h timepoint). In the case of PipX^ox^, PSII quantum yield was less affected, differing from that of Ptrc in that IPTG addition had a small positive effect, detectable after 4 h of its addition and maintained during the length of the experiment (Figure 3C). In good agreement with the apparent PSII quantum yield, the analysis of oxygen evolution after the first 24 h of IPTG induction indicated that PipX^ox^ was only slightly affected, while RelQ^ox^ and to greater extent PipY^ox^ were drastically impaired, with hardly any production or consumption of oxygen during the corresponding light and dark phases of the experiment (Figure 3D). Thus, the severe (PipY^ox^ and RelQ^ox^) or mild (PipX^ox^) impairment of photosynthetic activity after IPTG induction of the cultures correlated with the growth recovery (impaired or normal, respectively) on permissive media (Figure 2D).

Taking into account the roles of NtcA as an activator of *nblA* transcription (Luque et al., 2001; Espinosa et al., 2007) and of PipX as NtcA coactivator (Espinosa et al., 2006, 2014) in *S. elongatus*, it is likely that chlorosis and cell arrest at PipX^ox^ cultures is induced by co-activation of *nblA* and other NtcA-dependent genes. However, the mechanism by which an excess of PipY strongly induces chlorosis remains largely obscure and we wondered whether the resemblances between PipY^ox^ and RelQ^ox^ phenotypes may provide some clues.

### Cell length phenotypes induced by overexpression of PipX, PipY, or RelQ

Independent reports from us and others showing that overexpression of either PipY or RelQ resulted in elongated cells (Hood et al., 2016; Labella et al., 2017) prompted us to compare the appearance of Ptrc, PipX^ox^, PipY^ox^, and RelQ^ox^ cells by confocal microscopy. Comparisons were performed at 0, 24, and 48 h after IPTG induction and the results are illustrated in Figure 4.

The results confirmed the elongated phenotypes of PipY^ox^ and RelQ^ox^ cells, and showed that the long-cell phenotype is detected earlier at PipY^ox^. However, at the end of the experiment, RelQ^ox^ cells were at least as long as PipY^ox^ cells. Interestingly, PipX^ox^ cells were smaller, thus establishing a clear phenotypic difference with both PipY^ox^ and RelQ^ox^ and further suggesting that PipX and PipY may have opposite roles in the context of cell elongation or cell division processes.

We previously speculated that perturbations of the amino/keto acid pool by changes in PLPBP levels may result in the accumulation of metabolic signals that would control cell size in cyanobacteria (Labella et al., 2017). PipX overexpression, altering the interplay with nitrogen regulators and increasing NtcA activity (Espinosa et al., 2007), would also affect the amino/keto acid pool and it is thus possible that key metabolites controlling cell size may be differentially altered in PipX^ox^ and PipY^ox^ cells. In this context, the possibility that PipX and PipY may interfere with each other’s functions was previously suggested by the expression patterns of differentially expressed genes in single and double *pipX* and *pipY* null mutants (Labella et al., 2017).

To explore the possible involvement of 2-OG in cell size regulation, we measured cell length in nitrogen-deprived cultures of *S. elongatus*, conditions in which 2-OG accumulates (Muro-Pastor et al., 2001; Forchhammer and Selim, 2020). However, no significant cell length differences were found between growing cultures and growth-arrested cultures deprived of nitrogen for 72 h (Figure 4B and Supplementary Figure S6A), arguing against 2-OG levels playing a role in cell size.

A simple correlation between protein levels and cell size in *S. elongatus* was previously observed with the global transcriptional regulator RpaB (Moronta-Barrios et al., 2013). However, *pipX* or *pipY* null mutants show no cell size defects (Labella et al., 2017 and Supplementary Figure S6B), thus ruling out a simple correlation between PipX or PipY protein levels and cell size (Labella et al., 2020a,b; Tremiño et al., 2022). PipX and/or PipY overexpression may have indirect effects on cell size due to metabolic perturbations, or they may alter protein–protein interactions involved in cell division. Since both proteins have purely regulatory functions and their corresponding genes are linked to the cell division gene *sepF* in cyanobacteria (Labella et al., 2017), we favor the later possibility as a working hypothesis.

### PipY stimulates production of polyphosphate

PolyP is a linear polymer of orthophosphates that plays complex and not well understood roles in the most diverse organisms (Bondy-Chorney et al., 2020; Sanz-Luque et al., 2020). PolyP accumulation is regulated under a variety of stress conditions in the different systems studied and could thus potentially be triggered by stress associated to overexpression of proteins inducing cell arrest. In *S. elongatus* overexpression of RelQ results in longer cells that accumulate large amounts of polyP (Hood et al., 2016). Interestingly, a connection between polyP accumulation and cell size have also been reported for *Synechococcus* OS-B and *Pseudomonas aeruginosa*, where the absence of the synthesizing enzyme polyP kinase (PPK) resulted in shorter cells (Fraley et al., 2007; Gomez-Garcia et al., 2013). To get further insights into polyP accumulation and its possible connection with cell size in *S. elongatus* we next compared polyP accumulation in our panel of overexpressing strains.

In cyanobacteria, quantification assays based on exopolyphosphatase (Bru et al., 2017) are scarcely reproducible due to the high levels of phosphate in the culture medium and thus 4′6-diamidino-2-phenylindole (DAPI), typically used to stain DNA, is a much-preferred option (Tijssen et al., 1982; Sanz-Luque et al., 2020). Since excitation with wavelengths greater than 400 nm provides significant distinction between DAPI associated with DNA or with polyP, optimized protocols for DAPI staining and subsequent visualization by confocal microscopy have been validated (Aschar-Sobbi et al., 2008; Hood et al., 2016; Christ et al., 2020).

**Figure 5:**
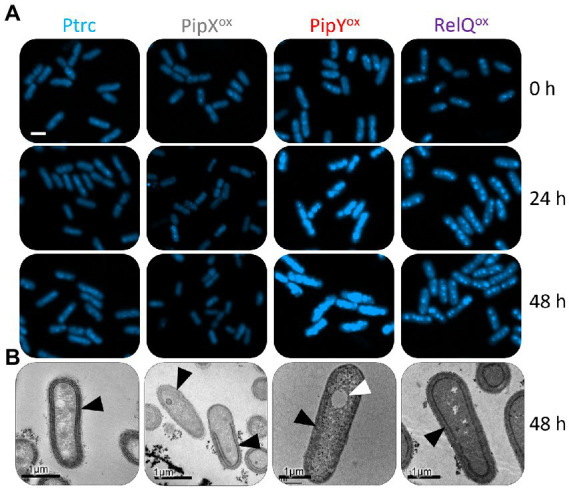
PipY^ox^ induces giant polyP granules. **(A)** Confocal micrographs showing DAPI-polyP staining and **(B)** thin-section transmission electron microscopy images of cells growing with IPTG for the indicated number of hours. White scale bar, 2 μm. Black arrowheads point toward the thylakoid membranes and white arrowheads to the atypical granular structures. Other details as in Figure 2.

Comparison of DAPI-stained cells from non-induced cultures (Figure 5A, time 0) showed that cells from RelQ^ox^ or (to a larger extent) PipY^ox^, but not from PipX^ox^, produced significantly higher signals. It is worth noting that promoter leakage from the P_trc_::*pipY* construct results in just a 3–4 fold increase of the levels of PipY in *S. elongatus* (Labella et al., 2017), indicating that the stimulatory effect of PipY on polyP granules, greatly magnified in the presence of IPTG, can take place close to normal intracellular levels of PipY. Importantly, IPTG addition did not alter the pattern of DAPI signals from PipX^ox^ or Ptrc cells, but it did significantly increase signals for RelQ^ox^ and, to a much greater extent, for PipY^ox^, where the DAPI signal often completely occupied the cytoplasm (24 and 48 h). The size of the giant DAPI-staining areas in PipY^ox^ cells was still dependent on the levels of the extracellular phosphate, as shown by using dipotassium phosphate (K_2_HPO_4_) concentrations above and below those contained in the standard BG11 medium (Supplementary Figure S7).

The extreme phenotype of the PipY^ox^ DAPI-stained cells prevented the use of the same visualization settings to compare numbers or sizes of polyP granules between PipY^ox^ and the other strains. Nevertheless, visual comparison clearly indicated that the number and size of polyP granules per cell were highest in PipY^ox^.

Transmission Electron Microscopy (TEM) was next used to visualize morphological changes in cellular structures and get further phenotypic insights into PipX^ox^, PipY^ox^, and RelQ^ox^ overexpressing cells after 48 h of IPTG induction. In complete agreement with the bleaching of PipX^ox^, PipY^ox^, and RelQ^ox^ cultures and the different severity of their phenotypes regarding pigmentation, loss of viability, and photosynthetic quantum yield, PipY^ox^ appears to have the most drastic destruction of the thylakoids. Consistent with the extreme phenotype revealed by DAPI staining, atypically large granular structures were found only in PipY^ox^ cells (Figure 5B and Supplementary Figure S4).

**Figure 6:**
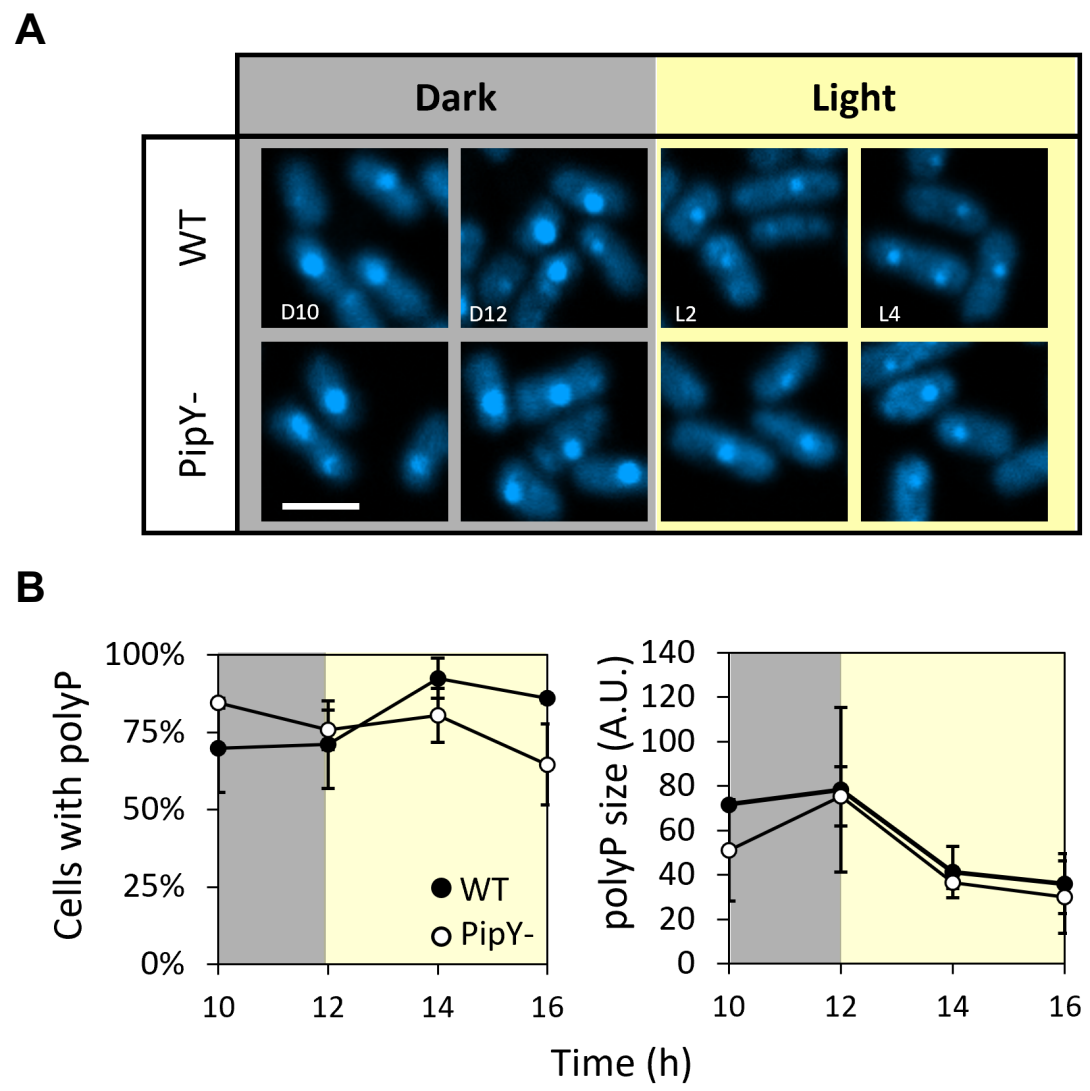
Inactivation of *pipY* does not impair the diurnal regulation of polyP accumulation. **(A)** Confocal micrographs showing DAPI-polyP staining of cells at dark (D10 and D12) and light (L2 and L4) timepoints during the L/D cycle. Cultures were trained in 12 h light/12 h dark cycles for 3 days. White scale bar, 2 μm. **(B)** Percentage of cells with at least 1 polyP granule (left panel) and representation of granule size (right panel) as detected area in arbitrary units. Data are presented as mean and error bars (standard deviation) of two biological replicates.

To get further insights into the role of PipY in polyP accumulation, we investigated the impact of the absence of PipY in the dynamics of the formation of polyP granules, which are more easily visualized in *S. elongatus* cells during darkness (Seki et al., 2014; Hood et al., 2016). To this end, we trained WT and mutant cultures in 12 h light/12 h darkness cycles for at least 3 days and subsequently analyzed the number of polyP per cell as well as their size (Figure 6A).

As shown in Figure 6B, in both WT and *pipY* strains, at least one polyP granule could be detected in most (> 60%) of the cells sampled throughout the experiment, with no significant differences between them in either number or size. The size of polyP granules increased similarly at the dark period and then decreased at the beginning of the light period, with no significant differences between *pipY* and WT strains. Therefore, this approach failed to show the implication of PipY in the regulation of polyP granules size or number under the diurnal cycle conditions used here.

Taken together, our results show that polyP overproduction in *S. elongatus* is not triggered by cell growth arrest or by the chlorosis process and also argue against a causal link between polyP levels and cell size, but support the idea that high levels of polyP, as observed in PipY^ox^ and RelQ^ox^ cells, increase cell length. In addition, the results also suggest that PipY, but not PipX, could regulate an unknown step of the pathway leading to polyP accumulation and/or control of granule size.

### Pleiotropic phenotypes induced by overexpression of PipX, PipY, or RelQ

The results presented so far, summarized in Table 2, expand the repertoire of pleiotropic effects caused by dosage perturbation of the *pipX*, *pipY*, or *relQ* genes in *S. elongatus*, revealing a remarkable resemblance between the overexpression phenotypes of PipY and RelQ, and key differences with PipX^ox^. The phenotypic descriptions carried out here open the way to future investigations of the regulatory details involved in the induction of growth arrest, chlorosis, cell size alterations or polyP accumulation in cyanobacteria.

**Table 2:** Impact of PipX, PipY and RelQ protein overexpression in *S. elongatus* features.

Characteristic	PipX^ox^	PipY^ox^	RelQ^ox^
Growth	⇩	⇩	⇩
Cell viability	⇩	⇩	⇩
Cell size	⇩	⇧	⇧
Pigmentation	⇩	⇩	⇩
Apparent PSII quantum yield	⇧	⇩	⇧/⇩
O_2_ evolution	⇩	⇩	⇩
PolyP accumulation	=	⇧	⇧

The direction (⇧⇩) of the arrows refer, respectively, to the severity of changes and effects on the corresponding phenotypic features, in relation to Ptrc values. See Figures 2–5 for additional details.

In particular, the phenotypic features described here for PipY^ox^ cultures should help studies on the structure–function relationship at PipY/PLPBP or concerning the regulatory complexities of accumulation of giant polyP granules in *S. elongatus*. For instances, PipY^ox^ derivatives carrying mutations at functionally relevant or conserved residues (Tramonti et al., 2022; Tremiño et al., 2022) can be easily compared in the presence of IPTG and further analyzed for traits of interest.

### PipY overexpression, a novel approach for massive polyP accumulation?

The biological production of polyP is of great interest as an environmentally friendly fertilizer (Siebers et al., 2019), and a key component of wastewater treatment or enhanced biological phosphate removal (EBPR) (Barnard et al., 2017). Since these processes depend on the presence of bacteria that accumulate large quantities of polyP, multiple attempts to engineer bacteria with increased polyP yield have been carried out. These strategies, most of them carried out in *E. coli*, dealt with increasing the levels of polyP kinase (PPK), encapsulating it, or increasing phosphate transport (Kato et al., 1993; Keasling et al., 1998; Liang et al., 2017). However, massive polyP accumulation in a cyanobacterium is of greatest biotechnological interest and, in this context, *S. elongatus* has been shown to be an option for sustainable wastewater treatment (Samiotis et al., 2021).

Recently, site-directed mutagenesis at the *E. coli ppk* gene followed by a genetic screening identified point mutations, apparently all targeting oligomerization residues, that dramatically increased polyP levels *in vivo* even in the absence of stress and, surprisingly, had normal levels of activity *in vitro* (Rudat et al., 2018). Notwithstanding the biotechnological potential of these *ppk* mutations, the study indicated an important regulatory complexity in the context of polyP production that is worth being investigated. In line with this, our work provides additional genetic evidence of the complexity of the regulation of polyP levels and reveals an unexpected pathway for massive production of polyP, this time in a cyanobacterium.

### The effects of PipY or PipX levels on ppGpp accumulation argue against a causal relationship between ppGpp alarmone and polyP accumulation

**Figure 7:**
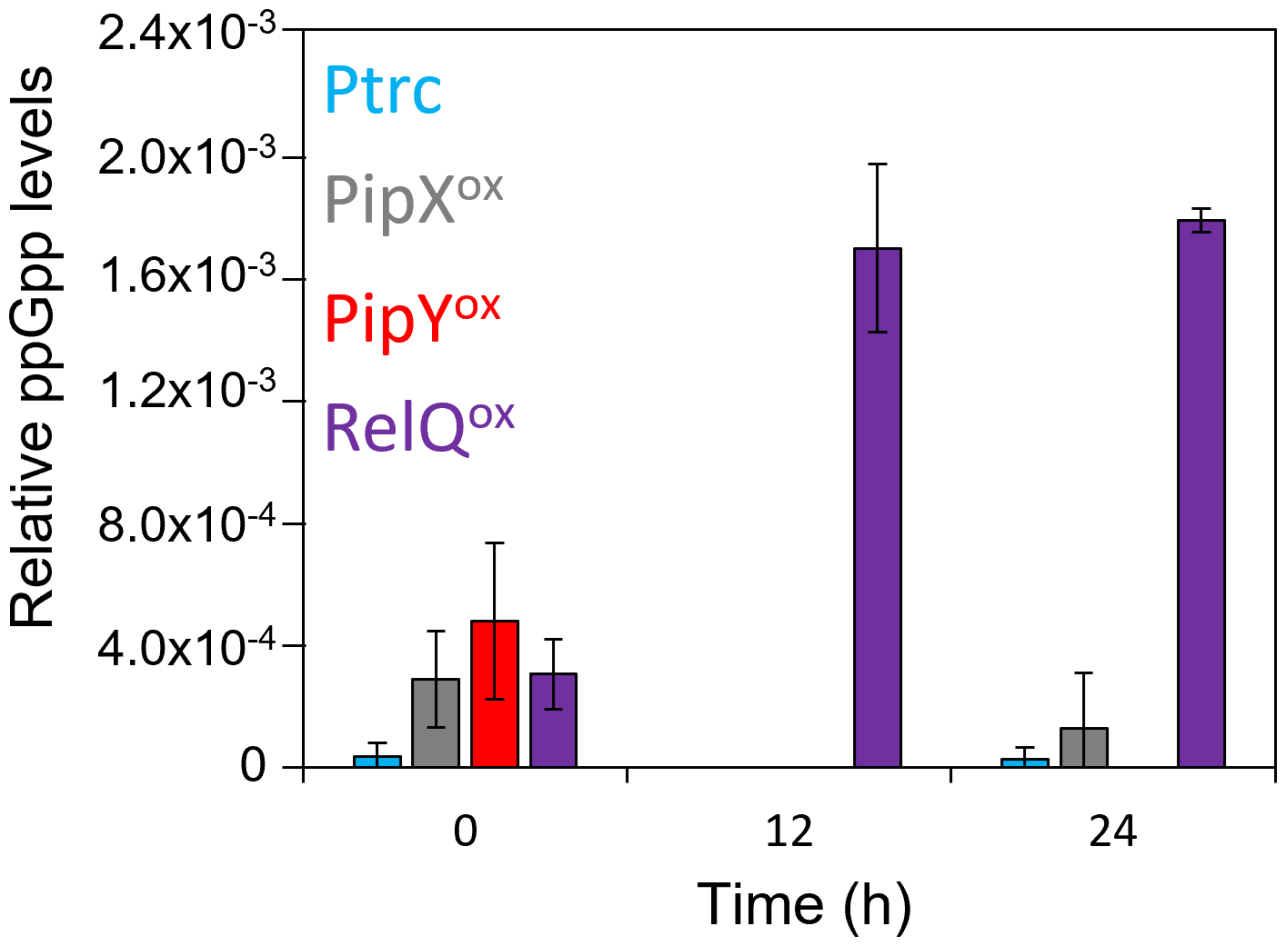
The stringent response is not induced by PipY^ox^ or PipX^ox^. Mass spectrometry analysis of relative ppGpp levels of strains grown under standard conditions with 50 μM IPTG for 0, 12, and 24 h. Relative ppGpp levels in each sample are calculated as the area under the curve (AUC) of the extracted ion chromatogram (EIC) for ppGpp and normalized to the AUC of the EIC for phosphoric acid (H_3_PO_4_). Data are presented as mean and error bars (standard deviation) of two biological replicates. Labels and data points are colored to indicate the strains as follows: Ptrc-light blue, PipX^ox^-gray, PipY^ox^-red, RelQ^ox^-purple.

We wondered whether PipX^ox^ and particularly PipY^ox^ cultures, that so far shared more phenotypic features with RelQ^ox^, could have induced the stringent response. With this in mind, we determined the levels of ppGpp and related nucleotides in cell extracts of strains PipY^ox^, RelQ^ox^, PipX^ox^, and of the Ptrc. We performed MS analysis using specific columns for extraction of phosphate-containing intracellular metabolites at times 0, 12, and 24 h after IPTG addition. No pppGpp or pGpp was detected in any of the samples. The results for ppGpp are summarized in Figure 7.

At time 0, the only strain where ppGpp levels were hardly detectable was Ptrc. It is worth noting that promoter leakage results in 3–4 fold increases in the levels of PipY or PipX (Labella et al., 2017; Cantos et al., 2019) and that the finding that ppGpp levels were higher in non-induced RelQ^ox^ cells than in the Ptrc control also agrees with production of significant basal levels of RelQ. Interestingly, ppGpp levels in PipY^ox^ and PipX^ox^ were as high as in RelQ^ox^ extracts at timepoint 0, suggesting that the increased PipY and PipX levels were somehow altering the regulation of the bifunctional (p)ppGpp synthetase/hydrolase Rel in *S. elongatus*. Given the importance of basal levels of ppGpp on the global control of gene expression in *S. elongatus* (Puszynska and O’Shea, 2017), the altered ppGpp levels of the non-induced cultures may have some physiological relevance in all three strains (PipX^ox^, PipY^ox^, and RelQ^ox^).

As expected for RelQ^ox^ cells, ppGpp levels greatly increased after IPTG addition. In sharp contrast, ppGpp levels in PipX^ox^ and PipY^ox^ extracts dropped to the levels of the Ptrc strain, being undetectable at the first timepoint taken after induction (12 h). Thus, none of the phenotypic similarities found in this work between RelQ^ox^ and PipX^ox^ or PipY^ox^ cells appear to require high levels of ppGpp.

Although accumulation of polyP is triggered by a variety of stresses in bacteria, pioneering work in *E. coli* suggested that (p)ppGpp directly stimulated polyP accumulation, presumably by inhibiting the exopolyphosphatase activity of the polyP degrading enzyme PPX (Kuroda et al., 1997). However, recent work challenged this view (Rudat et al., 2018) and showed that in *E. coli* polyP accumulation is not regulated by (p)ppGpp (Gray, 2019). In line with this, our results argue against a simplistic causal relationship between the intracellular levels of the ppGpp alarmone and those of polyP in *S. elongatus*.

## Concluding remarks

To gain further insights onto the functions of *S. elongatus* PipX and PipY and their interrelationships, we have performed a comparative analysis of the effects of overexpression of PipX, PipY, and RelQ on culture growth, cell viability, cell size, pigment content, photosynthesis activity, polyP accumulation, and ppGpp content. As a result, we have discovered a dramatic phenotype associated with high intracellular levels of PipY, the production of giant polyP granules. We also showed that polyP accumulation in *S. elongatus* is independent of the accumulation of the ppGpp alarmone. The production of giant polyP granules in *S. elongatus*, which are of biotechnological importance, opens the door to new experimental approaches and research lines in the study of PLPBP family members.

Regarding the involvement of PipX and PipY in common regulatory pathways, we found that the intracellular accumulation of PipX and PipY has opposite effects for some traits including cell length and apparent PSII quantum yield but also that excess of PipY is more deleterious, having a considerable impact on growth, viability, thylakoid membranes, apparent PSII quantum yield, and oxygen evolution. Finally, the specific dysregulation of polyP accumulation by excess PipY but not of PipX suggests that PipY could regulate an unknown step of the pathway leading to polyP accumulation and/or control of granule size.

In summary, this work expands our knowledge on the complexity of the regulatory interactions and signaling pathways in which PipX and PipY are apparently involved, providing new leads for future studies and for the biotechnological exploitation of results.

## Data availability statement

The original contributions presented in the study are included in the article/Supplementary material, further inquiries can be directed to the corresponding author.

## Author contributions

AL, JL, KF, KAS, and AC designed the research. AL, JL, and MB performed the research. AC wrote the initial draft of the manuscript with inputs from AL, KF, and KAS. All authors analyzed the data, contributed to the manuscript revision, and read and approved the submitted version.

## Funding

This work was supported by grants PID220-118816GB-I00 from the Spanish Government (MICINN) and VIGROB-126/21 from the University of Alicante to Asunción Contreras, PROMETEO/2021/057 to Francisco Martínez-Mojica from the Generalitat Valenciana, EXC 2124–390838134 of the DFG to Karl Forchhammer and Khaled A. Selim, the Federal Ministry of Education and Research (BMBF), the Baden-Württemberg Ministry of Science (PRO-SELIM-2022-14), 398967434-TRR261 from the DFG 832 and EXC2124 (CMFI) to Christoph Mayer. Antonio Llop was the recipient of a Santander-UA fellowship (BOUA/2021).

## Conflict of interest

The authors declare that the research was conducted in the absence of any commercial or financial relationships that could be construed as a potential conflict of interest.

## Publisher’s note

All claims expressed in this article are solely those of the authors and do not necessarily represent those of their affiliated organizations, or those of the publisher, the editors and the reviewers. Any product that may be evaluated in this article, or claim that may be made by its manufacturer, is not guaranteed or endorsed by the publisher.

## Footnotes

^1^ https://www.doubling-time.com/compute_more.php
