# Supplementary figures and images for: Pleiotropic effects of PipX, PipY, or RelQ overexpression on growth, cell size, photosynthesis, and polyphosphate accumulation in the cyanobacterium Synechococcus elongatus PCC7942
Source: Front Microbiol. 2023 Mar 16;14:1141775. doi: 10.3389/fmicb.2023.1141775 (PMC10060972; doi:10.3389/fmicb.2023.1141775)

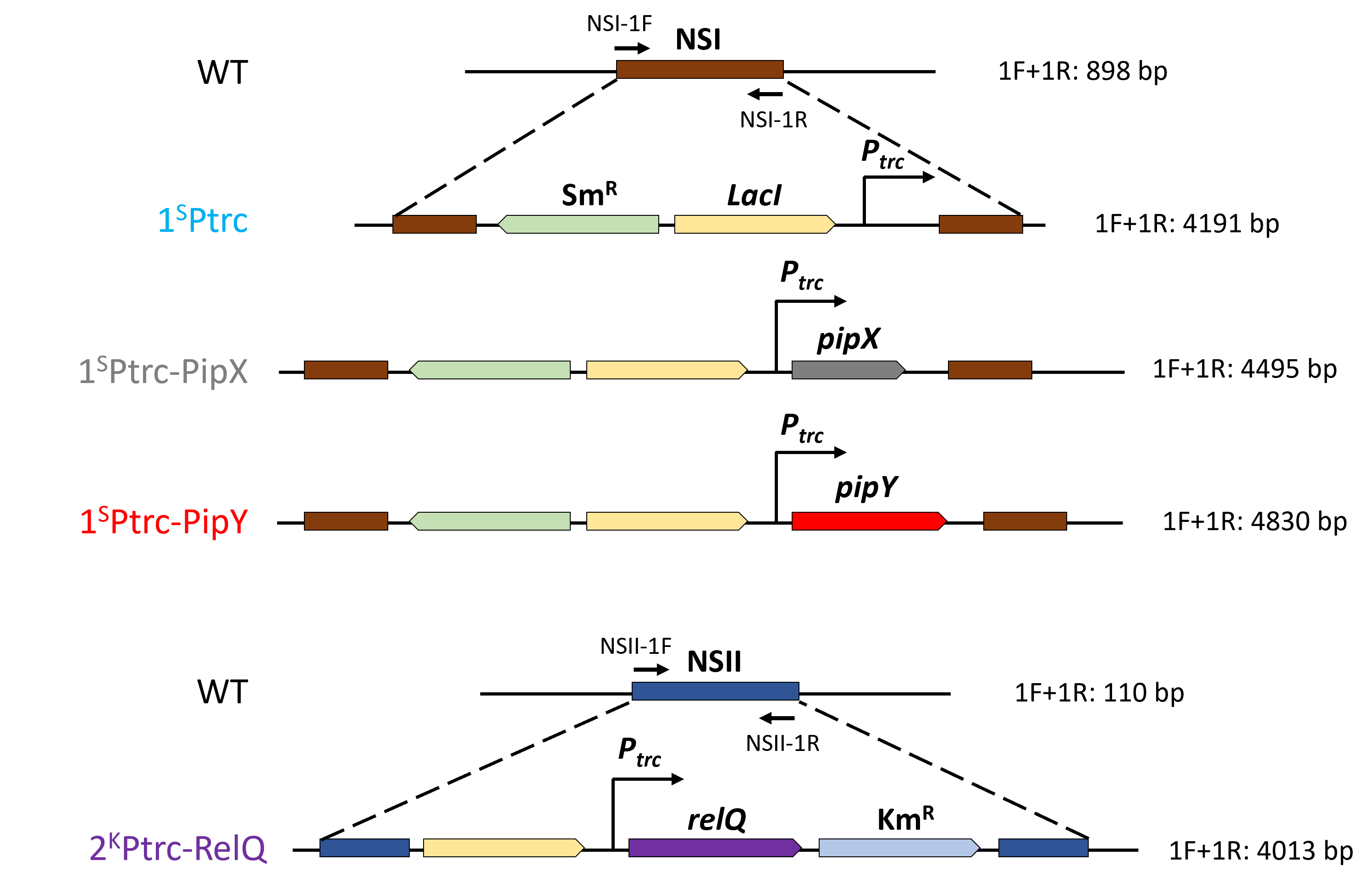

Supplement: Supplementary file 1 [file Image_1.TIF]

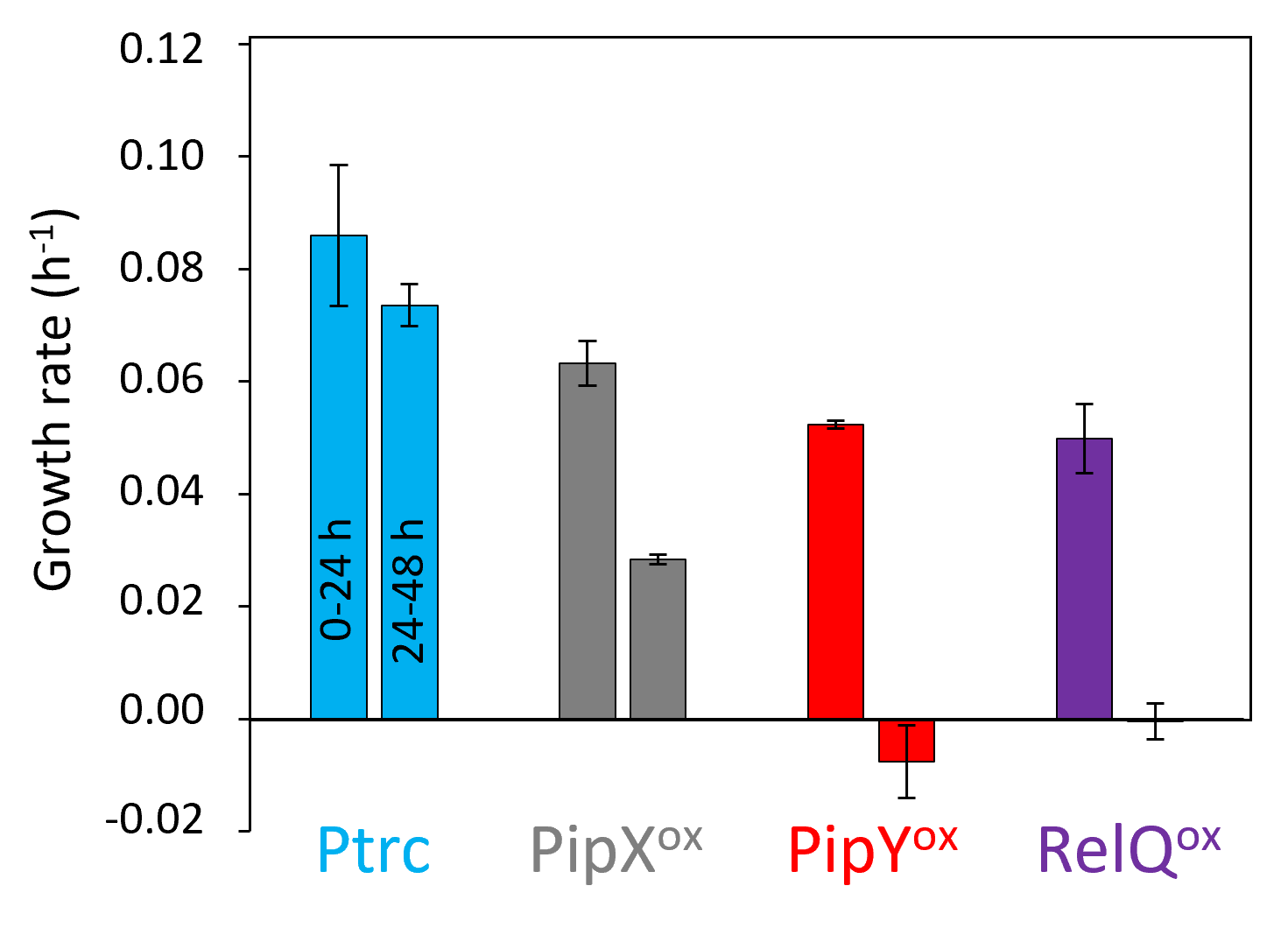

Supplement: Supplementary file 2 [file Image_2.TIF]

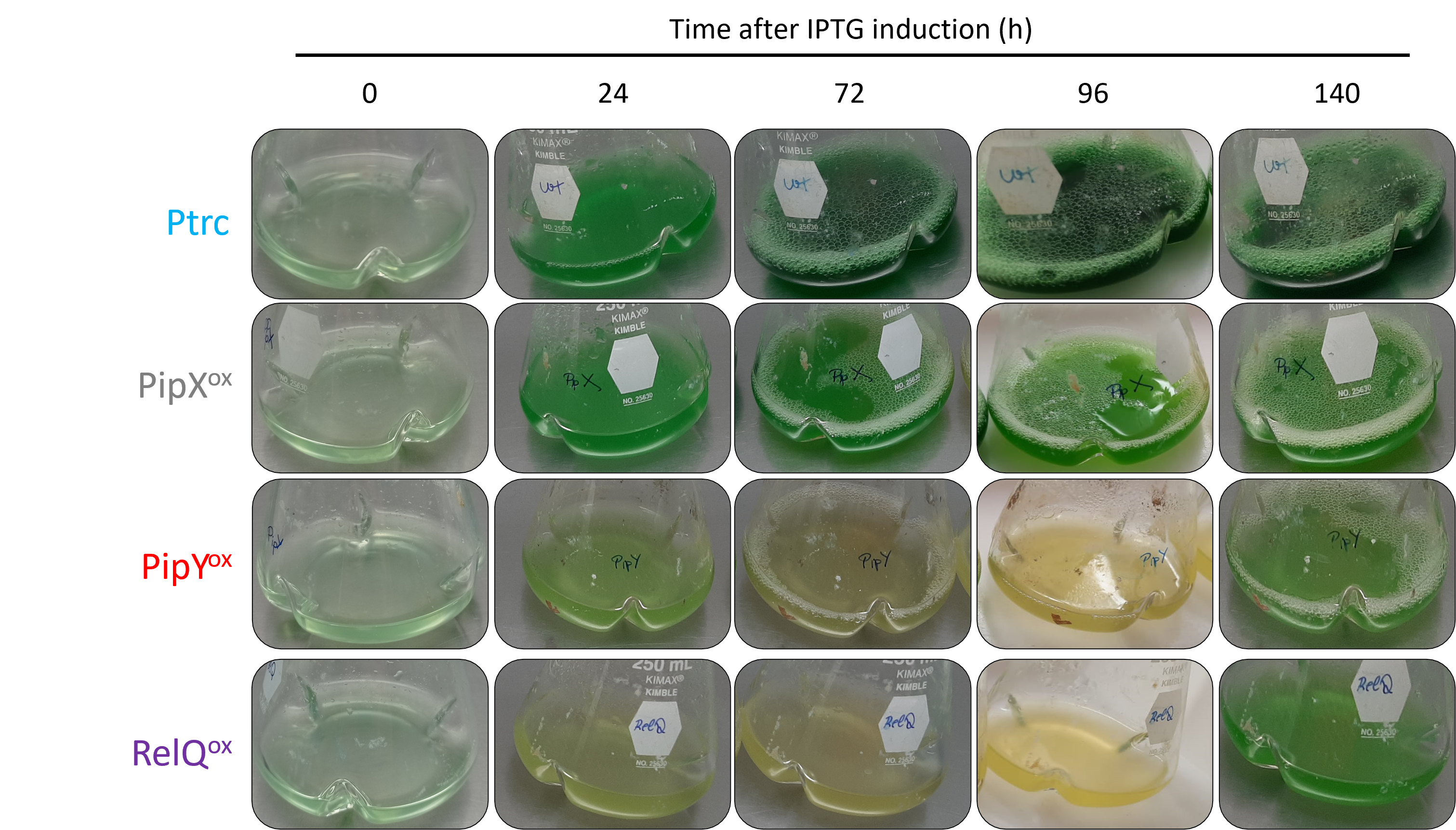

Supplement: Supplementary file 3 [file Image_3.TIF]

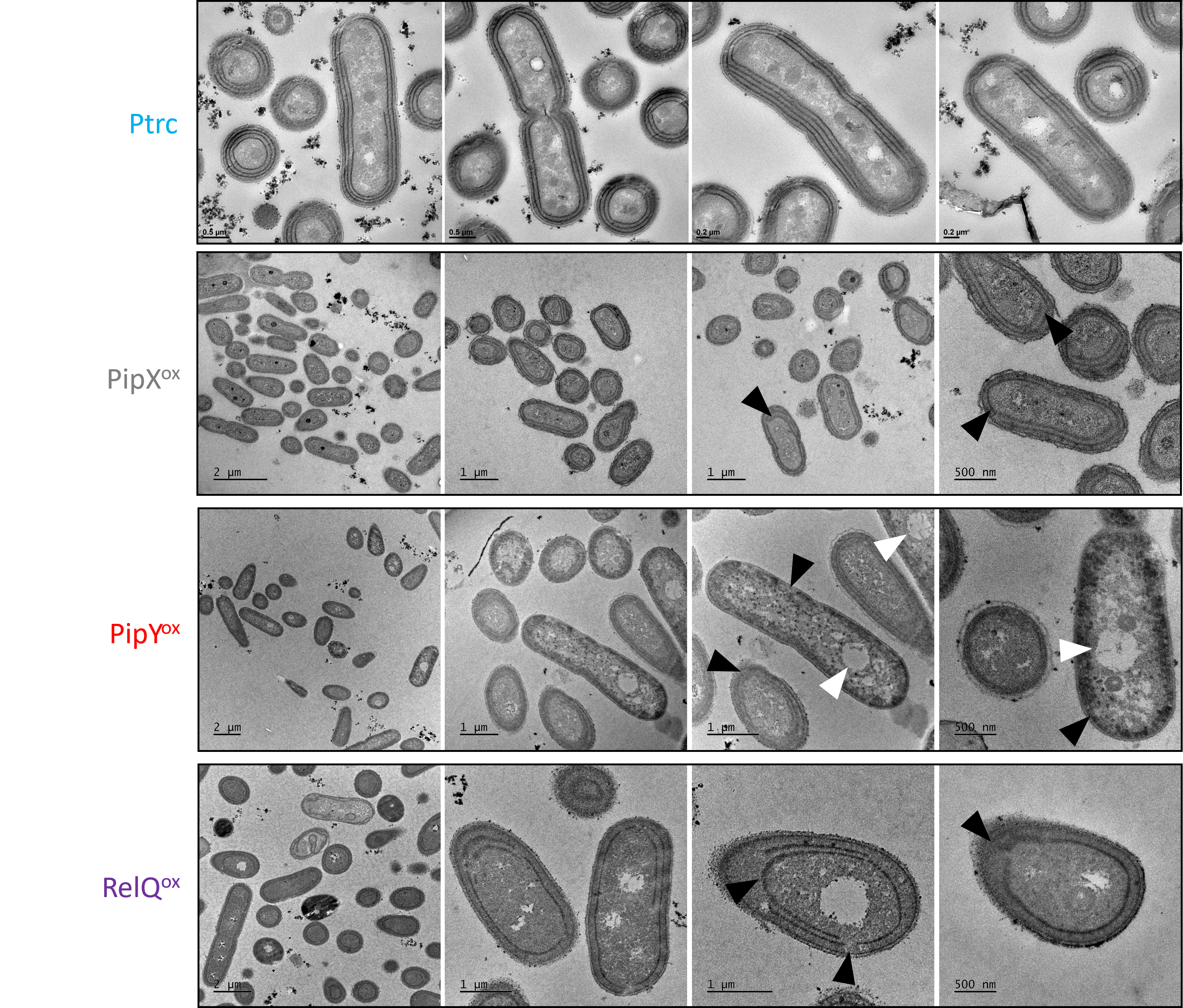

Supplement: Supplementary file 4 [file Image_4.TIF]

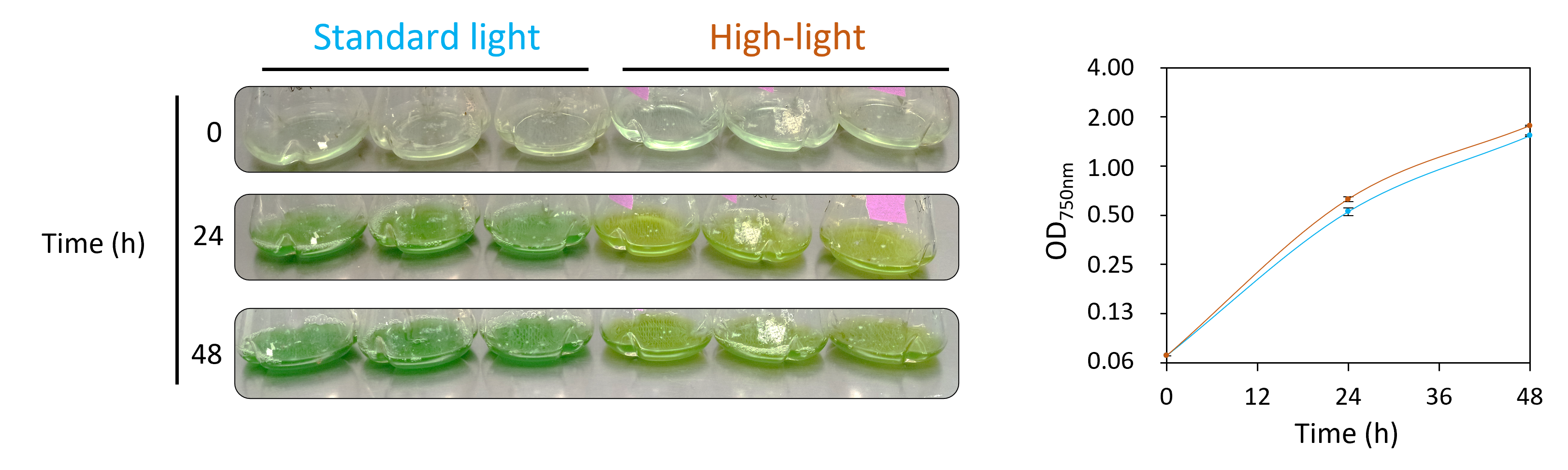

Supplement: Supplementary file 5 [file Image_5.TIF]

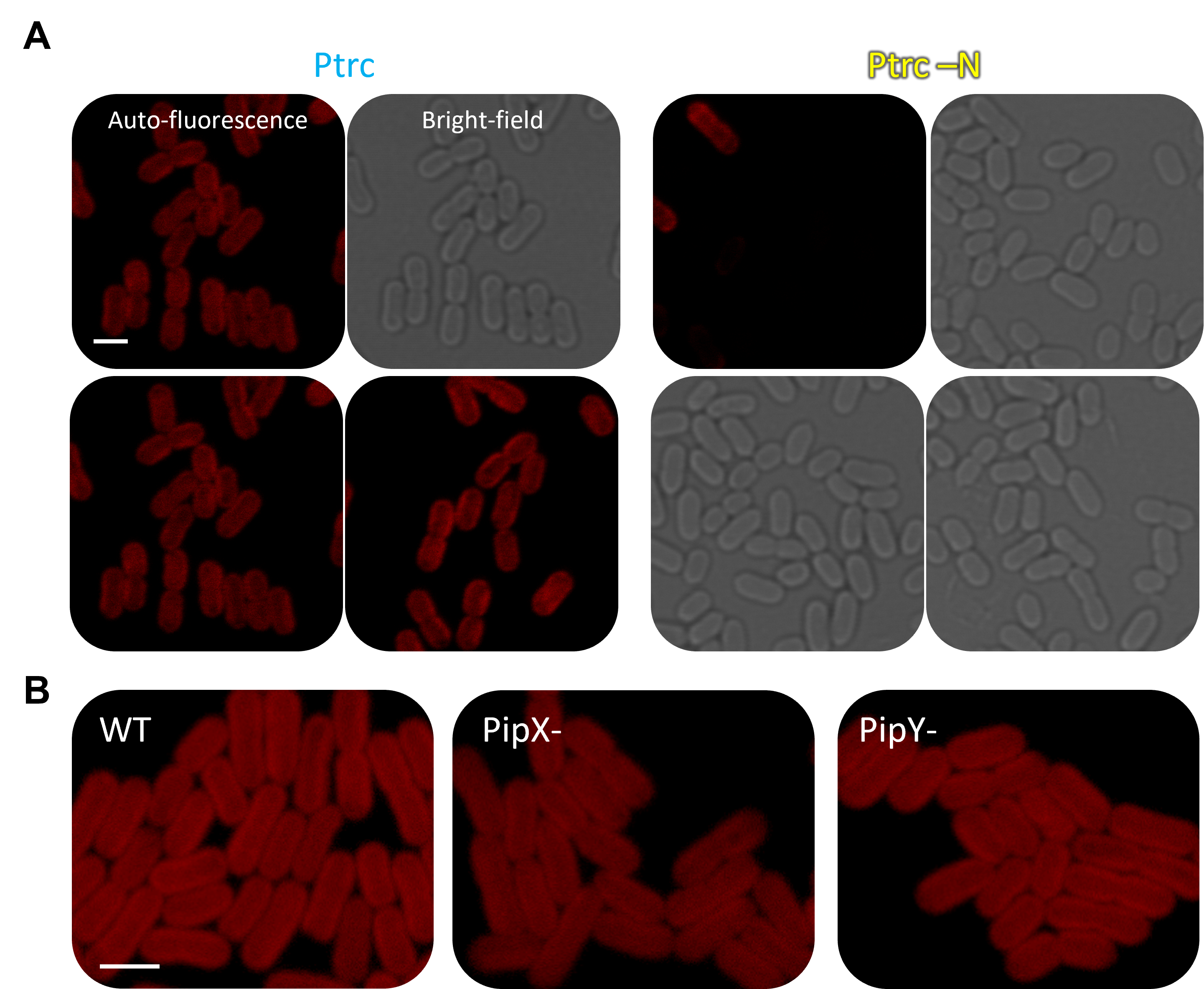

Supplement: Supplementary file 6 [file Image_6.TIF]

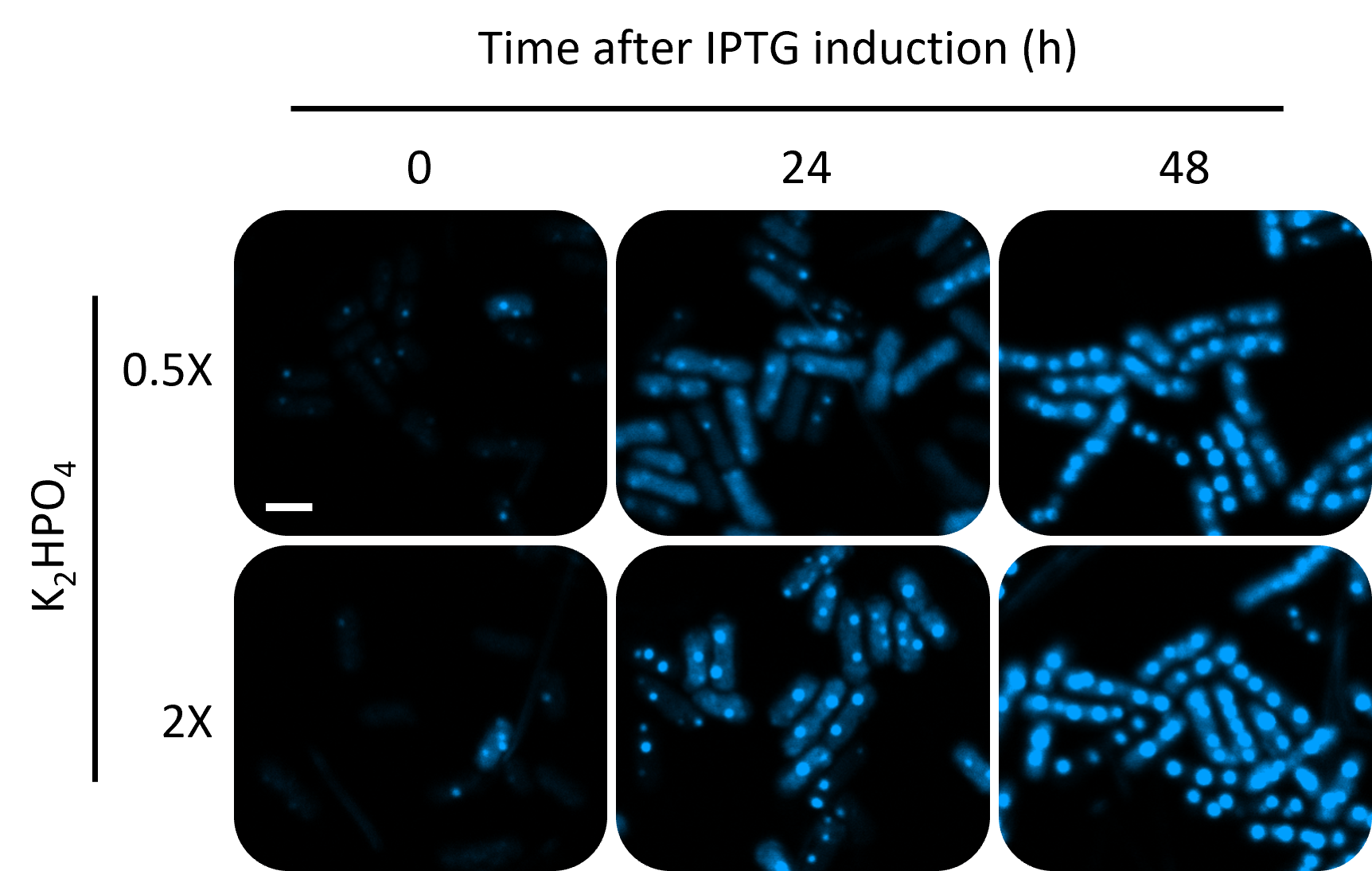

Supplement: Supplementary file 7 [file Image_7.TIF]
